# Unravelling Paclitaxel Resistance in Gastric Cancer: The Role of Small Extracellular Vesicles in Epithelial Mesenchymal Transition and Extracellular Matrix Remodelling

**DOI:** 10.3390/cancers17081360

**Published:** 2025-04-18

**Authors:** Giorgia Panzetta, Annalisa Schirizzi, Francesco Balestra, Maria De Luca, Nicoletta Depalo, Federica Rizzi, Angela Dalia Ricci, Giampiero De Leonardis, Claudio Lotesoriere, Gianluigi Giannelli, Rosalba D’Alessandro, Maria Principia Scavo

**Affiliations:** 1 Laboratory of Molecular Medicine, National Institute of Gastroenterology, IRCCS “S. de Bellis” Research Hospital, Via Turi 27, Castellana Grotte, 70013 Bari, Italy; giorgia.panzetta@irccsdebellis.it (G.P.); francesco.balestra@irccsdebellis.it (F.B.); maria.deluca@irccsdebellis.it (M.D.L.); 2 Laboratory of Experimental Oncology, National Institute of Gastroenterology, IRCCS “S. de Bellis” Research Hospital, Via Turi 27, Castellana Grotte, 70013 Bari, Italy; annalisa.schirizzi@irccsdebellis.it (A.S.); giampiero.deleonardis@irccsdebellis.it (G.D.L.); 3 Institute for Chemical-Physical Processes, Italian National Research Council (IPCF)-CNR SS Bari, Via Orabona, 70125 Bari, Italy; n.depalo@ba.ipcf.cnr.it (N.D.); f.rizzi@ba.ipcf.cnr.it (F.R.); 4 National Interuniversity Consortium of Materials Science and Technology (INSTM), Bari Research Unit, Via Orabona 4, 70126 Bari, Italy; 5 Medical Oncology Unit, National Institute of Gastroenterology, IRCCS “S. de Bellis” Research Hospital, Via Turi 27, Castellana Grotte, 70013 Bari, Italy; angela.ricci@irccsdebellis.it (A.D.R.); claudio.lotesoriere@irccsdebellis.it (C.L.); 6 Scientific Direction, National Institute of Gastroenterology, IRCCS “S. de Bellis” Research Hospital, Via Turi 27, Castellana Grotte, 70013 Bari, Italy; gianluigi.giannelli@irccsdebellis.it

**Keywords:** gastric cancer, extracellular vesicles, EMT, ECM

## Abstract

**Background:** Gastric cancer (GC) is a highly aggressive disease often complicated by resistance to chemotherapy agents like paclitaxel (PTX), which targets microtubules to induce apoptosis. Resistance arises through complex molecular mechanisms, including the overexpression of pro-angiogenic factors (VEGFA, ANG-2), activation of survival pathways (PDGFRβ, PPARγ), and epithelial-mesenchymal transition (EMT) driven by proteins such as VIM, E-CAD, N-CAD, and FLOT-1. The extracellular matrix (ECM), regulated by COL1A1 and influenced by PPARγ, acts as a physical barrier to drug penetration. Small extracellular vesicles (sEVs) have emerged as critical mediators of intercellular communication and may influence these resistance pathways. **Methods:** This study investigated the role of sEVs isolated from metastatic GC patients treated with Ramucirumab and PTX. Patients were stratified by progression-free survival (PFS) into rapidly progressing (RP) and controlled disease (CD) groups. sEVs from these patients were applied to HCEC-1CT and HEPA-RG cell lines. Cell viability assays, gene and protein expression analyses, and bioinformatic studies were conducted to assess the impact of sEVs on resistance-related markers. **Results:** Results showed that sEVs from CD patients reduced the expression of markers associated with drug resistance, while sEVs from RP patients increased these markers, promoting angiogenesis, EMT, and ECM remodeling. These changes correlated with enhanced cell survival and resistance phenotypes. Bioinformatic analyses confirmed that sEVs modulate inflammation, ECM dynamics, and EMT pathways. **Conclusions:** In conclusion, sEVs from metastatic GC patients significantly influence chemoresistance and tumor progression. Targeting sEV-mediated signaling may offer novel therapeutic strategies to overcome resistance and improve treatment outcomes in gastric cancer.

## Simple Summary

Gastric cancer presents a significant therapeutic challenge, particularly due to the development of resistance to chemotherapeutic agents such as paclitaxel. This study explores the role of small extracellular vesicles (sEVs), nanoscale particles secreted by cancer cells, in mediating drug resistance. These vesicles are known to modulate cellular behavior and may facilitate the survival of malignant cells under chemotherapeutic stress. The research focuses on patients with metastatic gastric cancer, aiming to elucidate the molecular mechanisms by which sEVs contribute to paclitaxel resistance. By analyzing the specific biomolecules contained within these vesicles, the study seeks to identify key mediators of resistance. The ultimate goal is to uncover novel therapeutic targets that may enhance the efficacy of current treatments and counteract resistance pathways. Insights gained from this research have the potential to significantly improve clinical outcomes for gastric cancer patients by informing the development of more effective and tailored treatment options.

## 1. Introduction

Gastric cancer (GC) poses a significant health challenge, particularly because of its aggressive behaviour and limited treatment options when it becomes resistant to chemotherapeutic agents. One such drug, paclitaxel (PTX), which interferes with the microtubule architecture and induces apoptosis, is widely used in treating various cancers, including gastric cancer [1]. However, PTX resistance represents a modern-day obstacle. For this reason, understanding the molecular mechanisms behind this resistance is crucial in order to develop new therapeutic strategies [2]. PTX resistance in gastric cancer is not driven by a single mechanism but rather involves complex interactions between multiple signalling pathways, transcription factors, and extracellular matrix (ECM) components [3]. The role of extracellular vesicles (EVs) in mediating drug resistance is a critical area of cancer research through mechanisms such as drug sequestration, efflux, and the transfer of bioactive molecules that modulate cancer cell survival and proliferation. Understanding these mechanisms can aid in developing novel therapeutic strategies to overcome chemoresistance [4,5]. EVs represent a heterogeneous group of membrane-bound structures released from almost all cell types into the extracellular environment. They include small EVs (sEVs) (<200 nm), which originate from the endosomal compartment, and large EVs (lEVs) (>200 nm), which are generated right from the plasma membrane. According to the MISEV (Minimal Information for Studies of Extracellular Vesicles, 2023) guidelines, EVs should be classified according to physical characteristics, biochemical composition, or biogenesis conditions because of overlapping markers and sizes [6]. Among sEVs, they are critical for intercellular communication and contribute to processes such as cell adhesion, epithelial-mesenchymal transition (EMT), and malignant progression [7,8]. In this study, we examined the role of small extracellular vesicles (sEVs) in these processes, while pointing out that other intracellular structures, such as endosomes, which arise from distinct yet related biogenetic pathways and are not classified within the sEV family, also contribute to intracellular signaling pathways, including those involved in apoptosis and chemoresistance [9,10]. This study focuses exclusively on the role of sEVs. Despite their different localization, sEVs and endosomes perform distinct yet interconnected roles in cancer biology, underscoring the complexity of cellular communication in tumor progression. sEVs derived from drug-resistant cancer cells can transfer proteins such as P-glycoprotein (P-gp) or multidrug resistance-associated proteins (MRPs) to sensitive cancer cells. These efflux pumps actively expel chemotherapeutic agents from cancer cells, leading to a decreased in intracellular drug accumulation and reduced efficacy [11]. sEVs can alter the tumour microenvironment (TME) to favour drug resistance. They can also promote the recruitment and polarization of immune cells such as tumour-associated macrophages (TAMs), which secrete factors that support cancer cell survival and chemoresistance. Additionally, sEVs can mediate the release of pro-inflammatory cytokines and growth factors that enhance the survival and proliferation of cancer cells [12]. Moreover, sEVs have been shown to be able to encapsulate chemotherapeutic drugs, reducing their bioavailability and therapeutic effectiveness. For example, some cancer cells release sEVs that seize and neutralize doxorubicin [13], preventing it from reaching its intended intracellular targets [14].

Recent studies have identified several key factors contributing to PTX resistance in tumor cells. First, PTX-resistant cells have been shown to exhibit an overexpression of pro-angiogenic factors, including Vascular Endothelial Growth Factor A (VEGFA), which promotes tumor growth and metastasis. Expression of this factor can contribute to the resistance mechanism by improving the tumor’s vascular supply and promoting a more aggressive cancer phenotype and expression of Angiopoietin 2 (ANG-2), which is known to promote tumor growth by stimulating the formation of new blood vessels that help to sustain tumour growth and protect the tumor microenvironment from chemotherapy-induced damage [13,15]. Second-line therapy combining PTX with the anti-angiogenic agent Ramucirumab, a monoclonal antibody that blocks the VEGFR2 receptor by preventing its binding to VEGF, may be a promising way to overcome PTX-mediated resistance by improving the therapeutic outcome [16]. Second, a notable alteration observed in PTX-resistant cell lines is the increased expression of Platelet-Derived Growth Factor Receptor Beta (PDGFRβ) and Peroxisome Proliferator-Activated Receptor Gamma (PPARγ). PDGFRβ is a receptor tyrosine kinase involved in the regulation of cell proliferation, migration, adhesion, endocytosis, and angiogenesis [17]. Overexpression of PDGFRβ has been associated with PTX resistance in gastric cancer [18]. High PDGF levels could also be related to resistance mechanisms that activate alternative pathways to stimulate angiogenesis following therapy blocking the main VEGF/VEGFR2 pathway [19]. PDGFRβ signalling enhances tumor cell survival and promotes a more aggressive phenotype, which can contribute to the failure of chemotherapy [20], through mechanisms that involve PPAR-γ, a nuclear receptor that regulates genes involved in cell differentiation, lipid metabolism, and inflammation [21]. Studies have shown that PPAR-γ can modulate drug resistance in cancer cells, including gastric cancer, and when it is either downregulated or suppressed, this can lead to an increase in the survival of cancer cells even in the presence of PTX [22]. The involvement of these factors illustrates a web of interconnected pathways contributing to PTX resistance in gastric cancer. For example, the EMT, mediated by factors like Vimentin (VIM), can work to create a tumor microenvironment that is difficult to target with conventional chemotherapy. Upregulation of VIM has been linked to increased drug resistance, as the EMT does not only facilitate metastasis but also provides cancer cells with a survival advantage against chemotherapy. VIM expression is often associated with more aggressive and resistant tumor phenotypes [23].

Another a protein involved in the cellular processes connected to chemoresistance, like intercellular adhesion and or endocytosis, is Flotillin-1 (FLOT-1). The activation of PPARγ can modulate the expression of membrane proteins, including FLOT-1, thereby influencing processes such as endocytosis and cellular signaling. Moreover, the activation of PPARγ can enhance the expression of proteins involved in lipid transport and the formation of membrane microdomains, where FLOT-1 plays a crucial role [24]. The modulation of FLOT-1 expression and function through PPARγ and PDGFRβ may contribute to drug resistance. Alterations in PPARγ signaling can influence drug endocytosis and intracellular trafficking, while changes in PDGFRβ signaling may impact cell proliferation and cell response to pharmacological treatments. Understanding these mechanisms is crucial for developing targeted therapeutic strategies to overcome drug resistance [25,26]. Additionally, the ECM, with components like collagen1A1 (COL1A1), plays a crucial role in both protecting cancer cells and modulating drug response. An overexpression of COL1A1 in GC has been associated with enhanced drug resistance. The ECM produced by COL1A1 can act as a physical barrier, thereby reducing drug penetration and providing cancer cells with protective signaling cues [27]. The present study aimed to identify potential predictive markers of response to second-line Ramucirumab and PTX therapy through comparative analysis of sEVs from patients stratified by treatment duration. In addition, the longitudinal analysis of sEVs throughout treatment until disease progression provided the basis for the identification of some key elements of sEVs in determining treatment outcome. An in-depth analysis of the content of sEVs revealed a key role in factors involved in angiogenesis, EMT, and ECM, including their associated molecules such as PPAR-γ, PDGFRβ, ANG-2, VEGFA, VIM, E-CAD, N-CAD, FLOT-1, and COL1A1. The study employs experimental methods designed to detect modifications in genes, proteins, structural cells modulation, and the potential overcome resistance with further improving treatment outcomes.

## 2. Materials and Methods

### 2.1. Patients Groups, Serum Preparation and Storage

Serum samples were obtained from a cohort of 41 patients diagnosed with metastatic GC undergoing second-line therapy with Ramucirumab and PTX. This longitudinal analysis was focused on serum samples collected at critical time points: prior to the initiation of therapy and at the first infusion of each treatment cycle. The study received formal approval from the appropriate Ethics Committee (protocol N°139/c.e., 28 June 2017), and all patients provided written informed consent to the use of their blood samples in the research. The patients’ characteristics have been described in a previous study [28]. The sEVs assay was conducted on serum samples collected at defined intervals: the baseline level (T0), at the first radiological control in the third cycle of therapy (T3), and at the time of radiological and/or clinical progression of the disease (TTP). For patients demonstrating particularly robust responses to therapy, additional analyses were performed at intermediate time points corresponding to periodic radiological re-evaluations (T6, T9, T12). Most importantly, two distinct patient groups were delineated based on their clinical evaluation after three months of treatment: those exhibiting rapid disease progression (RP) and those demonstrating a controlled disease (CD), characterized by either a partial response or a stable disease. This detailed analysis highlights the rigorous and dynamic approach employed to assess therapeutic efficacy and progression markers in this study. Whole blood was collected from these patients in commercial tubes containing a separating gel and silica-based clotting activator capable of separating serum from the corpuscular part of the blood. To ensure the removal of cells, including platelets, the serum was subjected to centrifugation at 2000× *g* for 15 min in a refrigerated centrifuge. Immediately after centrifugation, the serum was carefully transferred into clean microcentrifuge tubes. To preserve sample integrity, the serum was aliquoted into 500 μL vials so as to avoid repeated freezing and thawing cycles, and was subsequently stored at −80 °C.

### 2.2. Isolation of Serum Derived Small ExtracellularVesicles

Serum samples from all enrolled subjects were processed for sEV extraction in accordance with established protocols reported in the literature [29]. Initially, serum aliquots were thawed and subjected to centrifugation at 3000× *g* for 15 min at 4 °C. The supernatants were then carefully transferred into clean tubes and centrifuged again at 3800× *g* for 15 min at 4 °C. Following this step, the serum samples underwent ultracentrifugation using a BECKMAN L-60 Ultracentrifuge (Brea, CA, USA) at 75,000× *g* for 1 h at 4 °C. Subsequently, the resulting supernatants were transferred into clean ultracentrifuge tubes for a second ultracentrifugation cycle performed at 100,000× *g* for 1 h and 30 min. The sEVs fraction was collected as a pellet, which was then resuspended in 200 µL of ultrapure water. For each sample, 50 µL of the sEVs suspension were immediately processed for dynamic light scattering (DLS) and transmission electron microscopy (TEM) analyses. The remaining sEVs suspension was stored at −80 °C for subsequent protein extraction and cell lines treatements. This rigorous, multi-step protocol ensured the isolation of sEVs, enabling precise downstream characterization and analysis.

### 2.3. Bioinformatics Analysis

The interactions among genes associated with the EMC and EMT modulated in GC were systematically predicted using the Search Tool for the Retrieval of Interacting Genes/Proteins (STRING database version 11.0), a robust platform for identifying gene and protein interactions.

### 2.4. Cell Culture

The HEPA-RG human hepatoma cell line (Thermo Fisher Scientific, Waltham, MA, USA) was cultured in a hepatocyte bullet kit medium comprising transferrin (0.5 mL), ascorbic acid solution (0.5 mL), HEGF (0.5 mL), recombinant human insulin (0.5 mL), hydrocortisone (0.5 mL), BSA/fatty acid-free supplement (10 mL), and GA-1000 (0.5 mL). All reagents were procured from Lonza Biowhittaker (Oslo, Norway). This medium was further enriched with 10% sEV-depleted fetal bovine serum (FBS) and 1% antibiotic-antimycotic solution containing 10,000 U/mL of penicillin and 10,000 U/mL of streptomycin, obtained from the same supplier. For sEVs treatments, proteins derived from patients with RP and CD were administered at a concentration of 20 μg/μL for durations of 8, 24, 48, 72, or 96 h, depending on the experimental objectives. These included cell viability assays, droplet digital PCR (ddPCR), and protein functionality studies. In protein functionality experiments, HEPA-RG cells were seeded in 6-well plates at a density of 1 × 10^6^ cells per well and stimulated upon reaching semi-confluence, then treatments were applied every 24 h. For RNA experiments, cells were seeded in 6-well plates at 0.5 × 10^6^ cells per well, reaching semi-confluence before receiving identical 24-h interval stimulations. In cell viability assays, HEPA-RG cells were cultured in 96-well plates at an initial density of 2 × 10^3^ cells per well. These cells were first stimulated 24 h post-seeding and subsequently every 24 h with sEVs. Additionally, HCEC-1CT normal colon cells, acquired from Thermo Fisher Scientific, were maintained using CoLo Up medium (Evercyte GmbH, Vienna, Austria) as per the manufacturer’s protocol. Cultures were sustained at 37 °C in a humidified atmosphere with 5% CO_2_ and supplemented with FBS depleted of sEVs, in accordance with supplier guidelines. Experimental treatments for HCEC-1CT cells mirrored those used for HEPA-RG cells in terms of seeding density, stimulation frequency, and methodological approaches.

### 2.5. RNA Extraction and Gene Expression Analysis Through ddPCR

The gene expression levels of Platelet Derived Growth Factor Receptor Beta (PDGFRB), Peroxisome Proliferator-Activated Receptor Gamma (PPARG), Phospho-Glyco-Protein (PGP), ANGPT2 that encode Angiopoietin 2 (ANG-2), Vascular Endothelial Growth Factor A (VEGFA), Collagen type I alpha 1 chain (COL1A1), CDH1 and CDH2 that encode E-cadherin and N-cadherin, respectively, Vimentin (VIM), and Flotillin 1(FLOT1) were evaluated by droplet digital PCR (ddPCR) in the HCEC-1CT and HEPA-RG cell lines and treated with sEVs derived from serum samples of GC patients at several points of the clinical disease progression: T0, T3, T9, TTP for CD and T0, T3 for RP patients, as well as in untreated cells by type used as a control (CTR). Briefly, RNA was extracted from frozen cell pellets stored at −80 °C, using RNeasy^®^ Mini Kit (QIAGEN, 40724 Hilden, Germany). All samples were maintained at −80 °C before reverse transcription. The RNA concentration was measured using a NanoDrop Lite (Thermo Fisher Scientific, Waltham, MA, USA), and an aliquot of 2 µg was transcribed into cDNA using the High Capacity cDNA Reverse Transcription Kit (applied biosystems by Thermo Fisher Scientific, Waltham, MA, USA) according to the following protocol: priming 15 min at 25 °C; reverse transcription 120 min at 37 °C; reverse transcription inactivation 5 min at 85 °C; and holding at 4 °C. Storage was conducted at −20 °C. Copy numbers per microliter of PDGFRB, PPARG, PGP, ANGPT2, VEGFA, COL1A1, CDH1, CDH2, VIM, and FLOT-1 cDNA were analysed in both treated and untreated cell lines and quantified by droplet digital PCR (ddPCR; QX200 Droplet Digital PCR System, Bio-Rad, Hercules, CA, USA) following the EvaGreen protocol. The reaction was performed in a total volume of 20 µL, including 20 ng of cDNA per sample, 10 µL of QX200™ ddPCR™ EvaGreenSupermix (Bio-Rad, Hercules, CA, USA), RNase-/DNase-free water (variable), and 100 nM primer SYBR^®^ Green Assay for ddPCR. Reagents were provided by Bio-Rad with the following assay ID numbers presents in Table 1. Additionally, Droplet Generation oil for EvaGreen (cat. #1864005) and ddPCR Droplet Reader Oil (cat. #1863004) were used in this assay. The cycling conditions were as follows: 1 cycle at 95 °C for 5 min; 39 cycles at 95 °C for 30 s; 39 cycles at 57 °C for 1 min; 1 cycle at 4 °C for 5 min; 1 cycle at 90 °C for 5 min; holding at 4 °C. Data processing was performed using QX Manager 2.0 Standard Edition (Bio-Rad).

### 2.6. Cell Viability of HCEC-1CT and HEPA-RG Cell Lines After Treatment with sEVs

Both the colon epithelial cell line HCEC-1CT and the hepatic cell line HEPA-RG were seeded into 96-well plates at a density of 2 × 10^3^ cells per well. After 24 h, the cells were treated with sEVs derived from the serum samples of CD and RP patients at baseline level (T0) and at different disease progression stages: T3, T9, and TTP for CD, and T3 for RP, incubated at 37 °C for 48 h, with patient-derived sEVs stimulation being administered every 24 h. In the same plates, untreated cells were seeded as controls. At the end of the 48 h of sEVs treatment, the cell lines were incubated with 20 µL of MTS tetrazolium reagent ([3-(4,5-dimethylthiazol-2-yl)-5-(3-carboxymethoxyphenyl)-2-(4-sulfophenyl)-2H-tetrazolium], CellTiter 96^®^ AQueous One Solution Cell Proliferation Assay, Promega, Madison, WI, USA) in 80 μL of medium for a total volume of 100 µL at 37 °C for 3 h with 5% CO_2_. The quantity of formazan product, measured by absorbance at 490 nm, was directly proportional to the number of living cells in culture. Absorbance was measured using iMark™ Microplate Reader (Bio-Rad, Hercules, CA, USA). To validate the results obtained with the MTS assay, the Crystal Violet assay was performed on HCEC-1CT and HEPA-RG cell lines. Briefly, after treatment with sEVs for 48 h, both cell lines were fixed with 4% paraformaldehyde (PFA) (Sigma-Aldrich, Milan, Italy) and incubated for 30 min. Following fixation, the medium containing PFA was discarded, and Crystal Violet dye was added to the cells and incubated for 10 min. The plates were then washed with cold water, and prior to solubilization, images of the cells were acquired using a Nikon Eclipse Ti2 confocal microscope in bright field at 20× magnification. Subsequently, the cells were solubilized with 1% SDS (Sigma-Aldrich, Milan, Italy), incubated for 30 min at room temperature, and washed again. Absorbance was measured at 595 nm using a PerkinElmer Victor Plate Reader (Lier, Belgium).

### 2.7. Proteins Evaluation

The protein expression analysis in sEVs, in HCEC-1CT and HEPA-RG cells after 72 h of treatment with sEVs, was performed with Western blotting. Target proteins studied included Platelet Derived Growth Factor Receptor Beta (PDGFRβ), Peroxisome Proliferator-activated Receptor Gamma (PPARγ), Phosphoglycoprotein (P-gp) or MultiDrug Resistance 1, (MDR1), Angiopoietin 2 (ANG-2), Vascular Endothelial Growth Factor A (VEGFA), Collagen type I alpha 1 chain (COL1A1), E-cadherin (E-CAD), N-cadherin (N-CAD), Vimentin (VIM), and Flotillin-1 (FLOT-1). Proteins were extracted from sEVs derived from CD and RP patient serum samples and pellets of the two frozen treated cell lines, using RIPA buffer with protease and phosphatase inhibitors (Thermo Scientific, Rockford, IL, USA). Protein concentrations were measured using the Bradford assay (Bio-Rad, Milan, Italy), and 25 µg of total protein extracts were separated on 4–20% polyacrylamide gels and transferred onto polyvinylidene fluoride (PVDF) membranes (Bio-Rad Laboratories, Milan, Italy). Membranes were incubated with primary antibodies against PDGFRβ, PPARγ, COL1A1, VIM, FLOT-1 (1:500 dilution, Cell Signaling Technology, Beverly, MA, USA), anti-glyceraldehyde-3-phosphate dehydrogenase (GAPDH) (1:1000 dilution, Cell Signaling Technology, Beverly, MA, USA), E-CAD, N-CAD (1:250 dilution, abcam, Cambridge, UK), VEGFA, CD63, CD81 (1:500 dilution, abcam, Cambridge, UK), P-gp (1:250 dilution, Santa Cruz, Santa Cruz, CA, USA), and ANG-2 (1:500 dilution, R&D Systems, 55413 Minneapolis, MN, USA). Following TBS-TWEEN washes, secondary antibodies were applied, and protein bands were detected using chemiluminescence (ECL, Bio-Rad, Italy). Signals were captured with a ChemiDoc Imaging System (Bio-Rad, Milan, Italy) and normalized to CD63 and CD81 for sEVs and GAPDH for both cell lines, then quantification was conducted via Image Lab 5.2.1 software (Bio-Rad, version 6.1).

### 2.8. Statistical Analysis

Overall survival (OS), which expressed the probability of survival analyzed by the non-parametric Kaplan–Meier method and the equality of survival between the RP and CD patient groups, was calculated for the two patient groups (RP and CD) and expressed as the median for each group.

The normality of the data distribution was assessed using the Shapiro–Wilk test, which guided the decision to apply parametric statistical methods. Group homogeneity was evaluated using the proportions test. Statistical analyses were conducted with two different software programs, depending on the dataset and type of comparison. Specifically, SPSS (IBM SPSS Statistics, version 26) was employed to perform one-way ANOVA for evaluating overall statistical differences between experimental conditions in the densitometric analysis of protein expression and in the absolute quantification of gene expression obtained via droplet digital PCR (copies/μL).When necessary, Bonferroni correction was applied to adjust for multiple comparisons. For comparisons involving only two independent groups, such as cell viability assays independent samples *t*-tests were conducted using GraphPad Prism, version 5.0 (GraphPad Software, San Diego, CA, USA). Results are reported as mean ± standard deviation (SD), with statistical significance thresholds defined as follows: * *p* < 0.05; ** *p* < 0.005; *** *p* < 0.0005.

## 3. Results

### 3.1. Patients Characteristics

The cohort analysed in this study comprised a total of 41 patients stratified into two groups based on their response to second-line therapy with Ramucirumab and PTX. Clinical and instrumental evaluations, including computed tomography (CT), were conducted quarterly in accordance with the Response Evaluation Criteria in Solid Tumors 1.1 (RECIST 1.1). The group of patients exhibiting Rapid disease Progression (RP group n = 16) consisted of individuals who demonstrated progression at the first assessment (progression-free survival [PFS] ≤ 3 months; n = 16). Conversely, the Controlled Disease (CD) group (n = 25) comprised patients who achieved either a stable disease state or a partial response at the initial clinical-radiological evaluation (PFS > 3 months) and continued therapy until disease progression or the onset of therapy-related toxicity. Table 1 provides detailed information on patients’ characteristics in both groups. The RP group exhibited a median PFS of 2.68 months and a median overall survival (OS) of 6.30 months. In contrast, the CD group showed a median PFS of 10.38 months and a median OS of 12.47 months, reflecting more favourable outcomes (Table 2).

### 3.2. Bioinformatic and Experimental Analysis of Tumour-Related Proteins in Gastric Cancer sEVs

**Figure 1:**
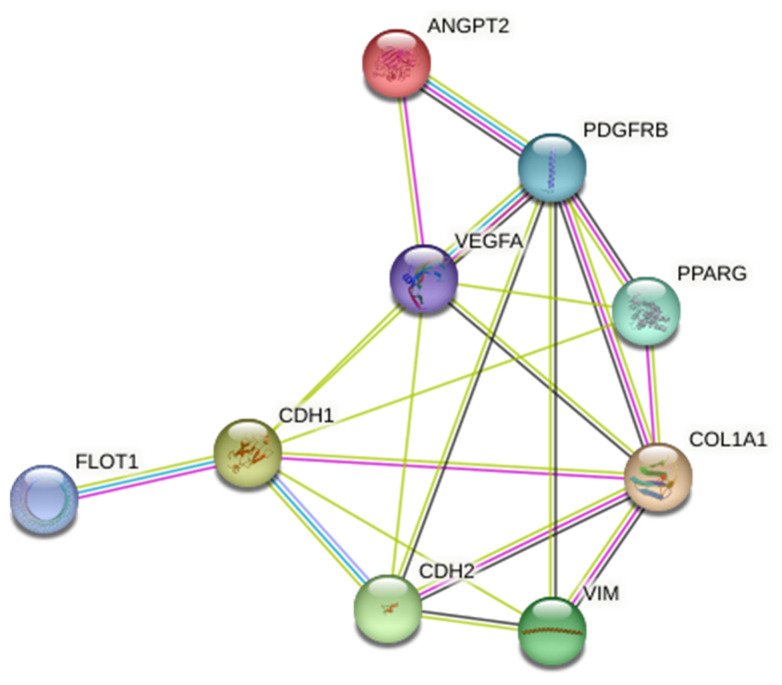
The interaction network was constructed using the STRING database, where nodes represent proteins and edges indicate predicted functional associations based on seven evidence sources: curated databases (light blue), experimental data (light pink), co-expression (grey), gene neighbourhood (yellow), gene fusions (orange), gene co-occurrence (blue), text mining (green), and protein homology (purple).

The interactions among several proteins encoded by genes identified through depth analysis of the literature were thoroughly examined using the STRING database. This preliminary bioinformatics step was essential to rationally select and confirm candidate proteins, which were subsequently validated through experimental analysis on patient-derived sEVs. Notably, the analysis highlighted the activation of PDGFRβ as a critical regulator, influencing PPARγ and ANG-2 proteins, specifically VEGFA, VIM, COL1A1, N-CAD, E-CAD, and FLOT-1, as illustrated in Figure 1, which are intricately involved in EMT and ECM modulation, respectively. These findings underscore the central role of PDGFRβ in orchestrating complex molecular pathways that contribute to the tumor cells progression and tissue remodeling. This analysis sheds light on the molecular underpinnings of sEVs-driven effects, offering valuable insights into potential therapeutic targets for mitigating disease progression.

**Figure 2:**
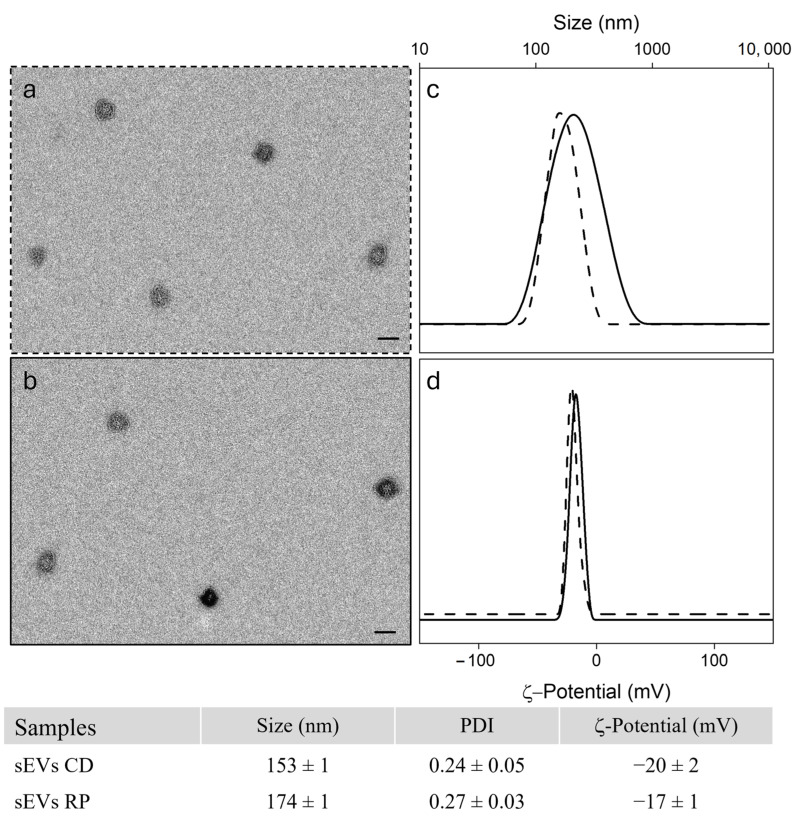
Representative TEM micrographs of sEVs extracted from serum of CD (**a**) and RP (**b**) patient groups (Scale bar 100 nm). Intensity size distributions by DLS (**c**) and ζ-potential values (**d**) for sEVs isolated from CD (dashed line) and RP (solid line) patients. DLS and ζ-potential data are reported in the table.

sEVs were isolated from serum samples obtained from 41 patients diagnosed with metastatic GC. Freshly isolated sEVs were comprehensively characterized for size, morphology, and surface charge. TEM revealed distinct circular, cup-shaped structures, consistent with sEVs morphology (Figure 2a,b). DLS analysis confirmed nanoscale dimensions, with average hydrodynamic diameters ranging from 150 to 175 nm across all samples (Figure 2c). Surface charge measurements showed negative ζ-potential values (Figure 2d), as summarized in the table in Figure 2.

**Figure 3:**
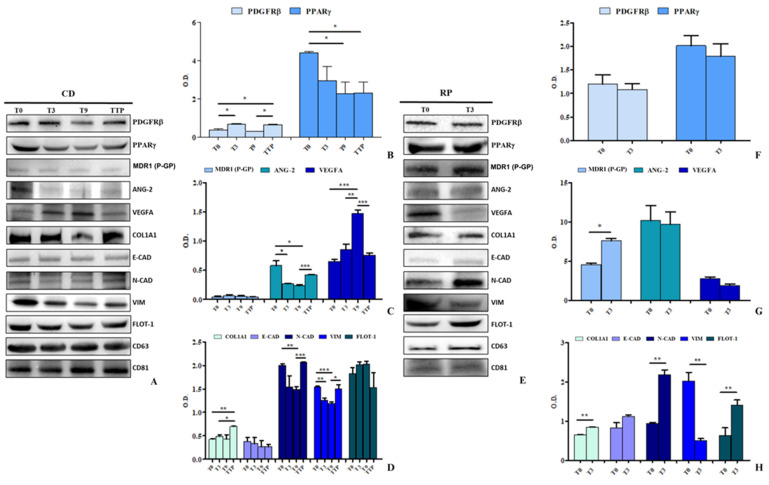
Evaluation of sEVs proteins isolated from serum samples derived from patients affected by GC responsive to the therapy (CD) and in rapid progression (RP) and treated with Paclitaxel baseline T0, before the first cycle, T3 before the 3rd cycle, T9 before the 9th cycle, and TTP, the progression time for CD and T0 before the first cycle and T3 the progression time. Representative Western blotting of different proteins (PDGFRβ, PPARγ, MDR1(P-gp), ANG-2, VEGFA, COL1A1, E-CAD, N-CAD, VIM, and FLOT-1) and housekeeping protein (CD63 and CD81) (**A**). Semiquantitative evaluation of the considered protein expression levels in sEVs obtained from patients CD after chemoterapy (**B**–**D**). The same treatment with sEVs derived from CD, Western blotting of the same and house-keeping proteins is reported (**E**). Semiquantitative evaluation of the considered protein expression levels in sEVs of RP patients after chemotherapy (**F**–**H**). The CD63 and CD81 proteins bands were used for the normalization of the proteins band for each subject. Statistical analysis was performed using one-way ANOVA with Bonferroni correction for multiple comparisons conducted using SPSS. Statistical significance is indicated as follows: * *p* < 0.05, ** *p* < 0.005, *** *p* < 0.0005. The uncropped bolts are shown in Supplementary Materials.

Western blot analysis was performed to assess the expression of key proteins involved in ECM remodelling and EMT, as identified through bioinformatics analyses. Representative Western blot images are shown in Figure 3A,E, while semiquantitative epiluminescence measurements are depicted in Figure 3B–H; the original Western blotting images can be found in Figure S1 of Supplementary Materials. The analyzed proteins include PDGFRβ, PPARγ, P-gp, ANG-2, VEGFA, COL1A1, E-CAD, N-CAD, VIM, and FLOT-1. Housekeeping proteins CD81 and CD63, which confirmed the presence of sEVs, were also examined (Figure 3A,E). This analysis revealed significant differences in protein expression between the RP and CD groups, highlighting the molecular characteristics of sEVs in metastatic GC. In the CD group, PDGFRβ, a key factor in tumor growth, survival, and angiogenesis, after an initial increase at T3, decreased significantly at T9 to later increase significantly at TTP (*p* < 0.05) (Figure 3A,B). In contrast, PPAR-γ, whose reduced expression is associated with more aggressive cellular phenotypes, was progressively reduced throughout therapy in the CD group (T0 vs. T9 and TTP, *p* < 0.05) (Figure 3A,B). Figure 3A,C illustrates protein expression related to drug response and angiogenesis in sEVs from CD patients. The levels of P-gp, actively involved in PTX resistance, remained consistently low regardless of disease progression. ANG-2, which promotes tumour angiogenesis, was significantly reduced between T0 and T3 and T0 and T9 (*p* < 0.05) but markedly increased between T9 and TTP (*p* < 0.0005). VEGFA, also involved in angiogenesis and replaced by Ramucirumab in case of therapy response, exhibited a significant increase in sEVs when patients responded to therapy (T0 vs. T3, *p* < 0.005; T0 vs. T9, *p* < 0.0005), but was drastically reduced upon disease progression (T9 vs. TTP, *p* < 0.0005). Further analysis of EMT and ECM markers (COL1A1, N-CAD, E-CAD, VIM, and FLOT-1) in sEVs from CD patients revealed significant modulation. COL1A1 levels increased during disease progression (*p* < 0.005), indicating ECM remodelling. N-CAD expression significantly decreased from T0 to T9 (*p* < 0.005) but sharply increased from T9 to TTP (*p* < 0.0005), mirroring VIM expression, which was significantly lower in responding patients (T0 vs. T3, *p* < 0.005; T0 vs. T9, *p* < 0.0005) but rebounded when therapy failed (T9 vs. TTP, *p* < 0.05). FLOT-1 expression remained almost unchanged (Figure 3A,D). These findings underscore the dynamic modulation of ECM remodelling and EMT-associated proteins in sEVs from CD patients, correlated with disease progression and therapeutic response. In Figure 3E, representative immunoblots performed on sEVs from patients experiencing rapid disease progression and immediate drug resistance at time T0 and after the first cycle of chemotherapy are shown (T3). In Figure 3F, the quantification of PDGFRβ and PPARγ expression indicates that their levels remained unchanged between these two time points, highlighting the ineffectiveness of the therapy. As expected, P-gp was present at higher levels in the sEVs of RP patients (compared to the CD group), likely playing a crucial role in conferring chemoresistance and promoting tumour progression. As presented in Figure 3E,G, a further significant increase in its expression was observed at T3 (*p* < 0.05), while no significant changes in the expression of ANG-2 and VEGFA were detected. EMT markers and COL1A1 were analyzed in sEVs, revealing a significant upregulation of both COL1A1 (involved in ECM) and N-CAD (involved in EMT) (*p* < 0.005). By contrast, no variation was observed in E-CAD expression, whereas VIM levels showed a significant decrease (*p* < 0.005). Finally, a strong significant increase of FLOT-1 expression was observed in the sEVs derived from RP patients (*p* < 0.005), confirming finally that FLOT-1 is indeed a marker of sEVs, is involved in the transport of pro-EMT factors, and promotes intercellular communication and the spread of pro-metastatic signals.

### 3.3. Impact of sEVs Derived from CD and RP Patients on HCEC-1CT and HEPA-RG Cell Lines Viability

**Figure 4:**
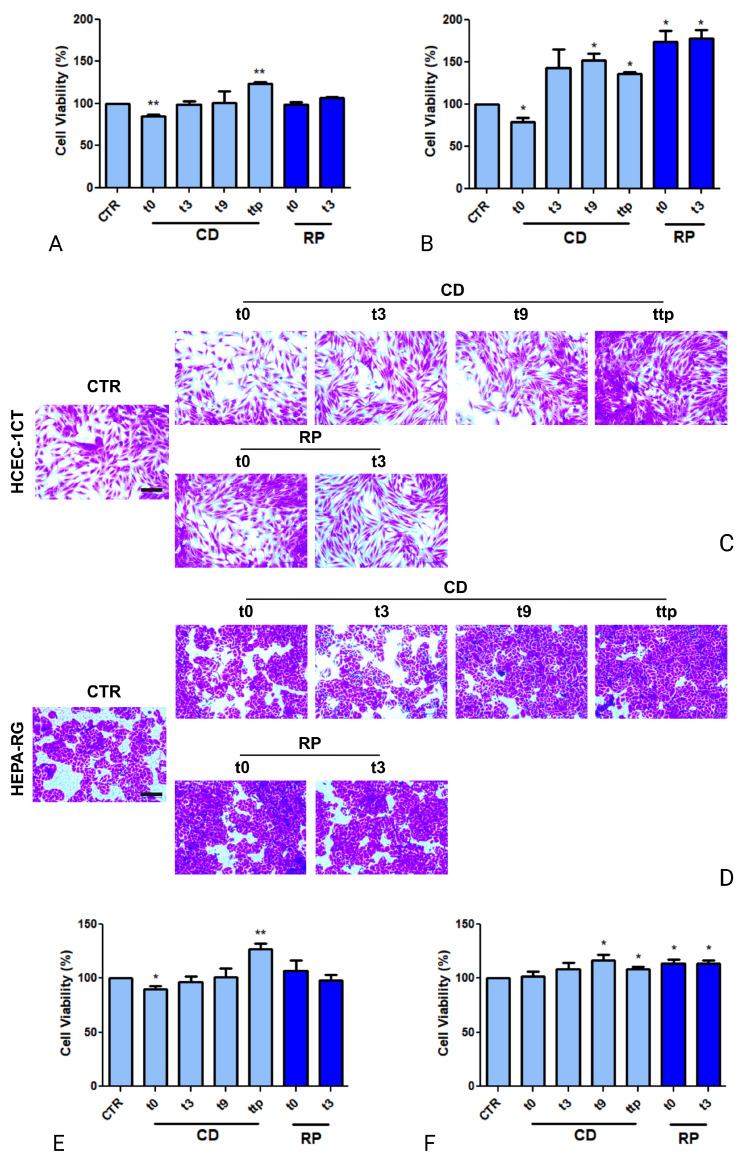
Graphs (**A**,**B**) display the viability of HCEC-1CT and HEPA-RG cells, respectively, treated with sEVs from GC patients. The data show that T0 sEVs from CD patients caused a decrease in cell viability for both HCEC-1CT and HEPA-RG cells compared to untreated CTR cells after 48 h of treatment, whereas TTP sEVs from CD patients are involved in the hyper-proliferation of both cell lines. The increase is not evident in the HCEC-1CT treated with RP sEVs from T0 and T3, while on the other hand, the increase is evident in HEPA-RG compared to the control when treated with RP sEVs. Crystal violet staining micrographs for HCEC-1CT (**C**) and HEPA-RG (**D**) detected by a phase contrast microscope (scale bar 100 μm) and corresponding analysis of total number of cells (**E**,**F**) after cell incubation with sEVs from CD and RP patients over the course of 48 h. Statistical analysis was performed using independent samples t-tests for comparisons between treated groups and control (CTR), conducted in GraphPad Prism. Statistical significance is indicated as follows: * *p* < 0.05; ** *p* < 0.005.

HCEC-1CT and HEPA-RG cell lines were treated with sEVs isolated from CD and RP patients at different time points (T0, T3, T9) for 48 h, followed by a cell viability evaluation by MTS assay. As shown in Figure 4, a significant reduction (*p* < 0.005) in HCEC-1CT cell viability was observed after the treatment of cells with sEVs derived from T0 of CD patients and then compared to untreated CTR cells. No change in viability, however, was observed after treatment with sEVs derived from T3 and T9 samples, while a significant increase was observed after treatment with sEVs derived from serum samples patients in progression, TTP (*p* < 0.005) (Figure 4A). Figure 4B demonstrates the effects of these sEVs on HEPA-RG cells. A notable decrease in viability (*p* < 0.05) was detected when these cells were treated with T0 sEVs from CD patients. Nonetheless, a striking hyperproliferation effect emerged when cells were treated with T9 and TTP sEVs from the same patients (*p* < 0.05), reinforcing the potential impact of the disease’s progression. Interestingly, a contrasting effect was observed in HEPA-RG cells treated with sEVs from RP patients. Unlike HCEC-1CT, HEPA-RG cells exhibited significant proliferation when exposed to T0 and T3 sEVs derived from RP patients (*p* < 0.05). This suggested a disease-specific influence of sEVs on different cell types, where RP-derived sEVs may enhance proliferation in hepatic cells. These findings indicated a crucial role of sEVs in modulating cell viability, with CD patient-derived sEVs exhibiting time-dependent effects consisting of initial suppression followed by enhanced proliferation during disease progression. Meanwhile, RP patient-derived sEVs appear to induce cell-specific proliferation, particularly in HEPA-RG cells. The data were validated using the Crystal Violet method, which serves as a crucial tool for confirming the number of viable cells remaining after treatment. Notably, Figure 4C reports a representative image of HCEC-1CT cells treated with small extracellular vesicles (sEVs) from controlled disease (CD) and rapidly progressing (RP) patients, while Figure 4D shows HEPA-RG cells treated similarly. As can be observed, a hyperproliferation is evident in both cell types at different time points considered, leading to disease progression when treated with sEVs of CD patients (Figure 4C,D). Furthermore, in the same figure C-D, hyperproliferation is observed in HEPA-RG cells treated with sEVs from RP patients, unlike HCEC-1CT cells, where this variation is not observed. Similarly, Figure 4E,F report the analysis of cellular color intensity, which follows the same trend as the MTS assay.

### 3.4. Analysis of Gene Expression in HCEC-1CT and HEPA-RG Cell Lines Treated with sEVs from CD and RP Patients Affected by Metastatic Gastric Cancer

**Figure 5:**
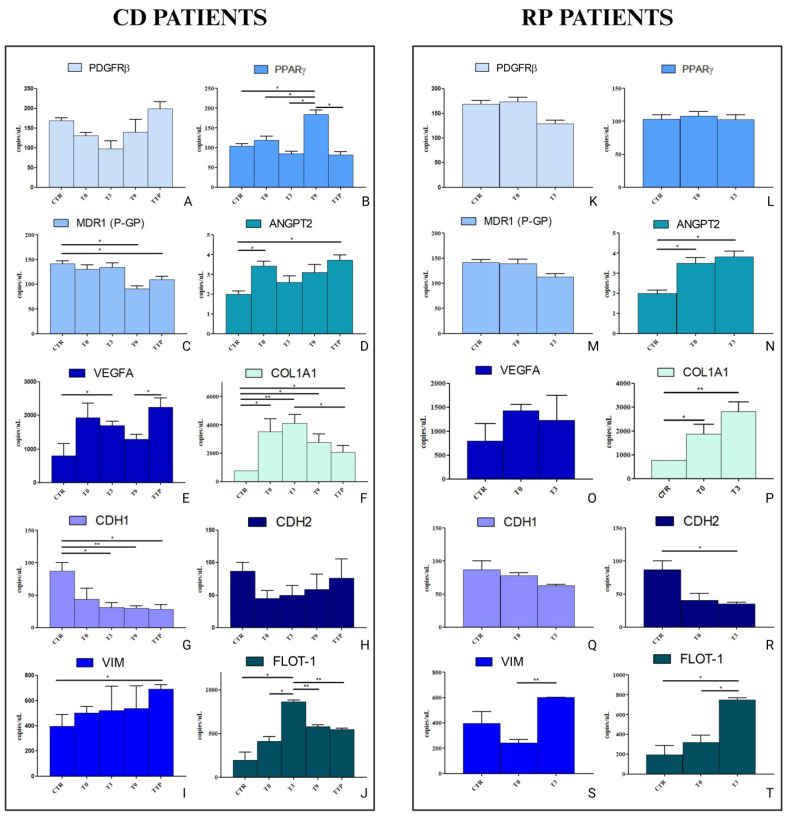
Droplet digital PCR analysis of PDGFRB, PPARG, MDR1(PGP), ANGPT2, VEGFA, COL1A1, CDH1(E-CAD), CDH2 (N-CAD), VIM, and FLOT-1 in HCEC-1CT cell line. The cells treated with serum-derived sEVs from patients with GC, Controlled Disease (CD), and Rapid progression (RP) at T0 (before the first cycle of PTX) before the third cycle (T3), before the ninth cycle (T9) and at progression (TTP), for CD, and T0 and T3 for RP patients. The value of copies/μL for PDGFRB is reported in (**A**), PPARG is reported in (**B**), MDR1(PGP) is reported in (**C**), ANGPT2 is reported in (**D**), VEGFA is reported in (**E**), COL1A1 is reported in (**F**), CDH1(E-CAD) is reported in (**G**), CDH2 (N-CAD) is reported in (**H**), VIM is reported in (**I**), and FLOT-1 is reported in (**J**) for CD patients, while the same genes are reported from (**K**) to (**T**) eeokfor RP patients. Statistical analysis was performed using one-way ANOVA with Bonferroni correction for multiple comparisons, conducted in SPSS. Statistical significance was defined as follows: * *p* < 0.05; ** *p* < 0.005.

Based on these bioinformatic findings, ddPCR was performed on HCEC-1CT and HEPA-RG cell lines treated for 48 h with sEVs derived from RP and CD patients. The expression of PDGFRB, PPARG, MDR1 (PGP), ANGPT2, VEGFA, COL1A1, CDH1, CDH2, VIM, and FLOT-1 was evaluated. Figure 5 summarizes the results for HCEC-1CT cells treated with sEVs from CD patients. PDGFRβ expression showed a downward trend from T0 to T9, thereby paralleling the active response to PTX. In contrast, cells treated with sEVs derived from CD-progressing patients exhibited a significant upregulation of PDGFRβ compared to controls (*p* < 0.05) (Figure 5A). Furthermore, our bioinformatic analyses revealed a link between PDGFRβ and PPAR-γ, which was further supported by the current gene expression data. PPAR-γ expression remained almost unchanged after treatment with sEVS from T0 and T3 patients, then increased significantly (*p* < 0.0005) after treatment with T9 sEVs, and finally decreased again (*p* < 0.05) following treatment with vesicles from patients in progression (Figure 5B). PGP expression decreased significantly (*p* < 0.05) in sEVs-treated cells of CD patients at T9 and then tended to increase at progression (Figure 5C). These findings underscore the critical involvement of P-gp in drug resistance mechanisms. Although ANGPT2 expression is generally low in epithelial cells, distinct changes were observed when cells were treated with sEVs derived from T0, T3, T9, and TTP samples of patients with controlled disease (CD). Notably, a pronounced increase (*p* < 0.05) in ANGPT2 expression was detected at T0 compared to CTR, followed by a decrease in its levels at T3 and T9 and a new increase at progression (Figure 5D). A regulatory effect was also observed in the modulation of VEGFA, a key factor in angiogenesis. The same trend was observed for VEGFA as described for ANGPT2, with a significant increase at progression, indicating a heightened angiogenic potential at this time point (Figure 5E). HCEC-1CT cells treated with sEVs from patients whose disease is therapeutically controlled still exhibit a significant upregulation of COL1A1 gene expression. This gene encodes a protein that is crucial in the composition of the ECM, widely known for being involved in chemotherapy resistance. As shown in Figure 5F, a statistically significant increase in COL1A1 expression (*p* < 0.05) is observed compared to the control (CTR) when cells are treated with sEVs derived from CD patients. Notably, this expression is even further enhanced when cells are treated with sEVs from patients at T3, displaying a highly significant increase compared to the control (*p* < 0.005). These findings underscore the potential role of sEVs in modulating ECM-related pathways, which may contribute to chemotherapy resistance even in therapeutically controlled disease states. A particularly striking finding is the relentless loss of CDH1 (E-CAD) expression, which persists despite the therapeutic response. This significant decrease is observed at all time points (*p* < 0.05 and *p* < 0.005, Figure 5G). These results highlight a progressive reduction in E-CAD expression, a key marker of epithelial integrity, suggesting a potential shift toward a more mesenchymal-like phenotype. This phenomenon may indicate an ongoing EMT process, which could contribute to tumor progression and therapy resistance, even in patients responding to treatment. The gene expression of CDH2 (N-CAD) remains unchanged across all time points, showing no significant differences compared to the control (Figure 5H). This stability in CDH2 expression may suggest that the loss of CDH1 (E-CAD) is not necessarily accompanied by a complete mesenchymal transition. Instead, it points to a more intricate regulation of cell adhesion dynamics in response to therapy, which may occur through the modulation of other EMT-related proteins, such as VIM and FLOT-1. These findings highlight the possibility of alternative mechanisms driving cellular plasticity, accentuating the complex interplay of adhesion and mesenchymal markers in tumour progression and therapy response. Regarding these two proteins, VIM and FLOT-1, a significant increase in expression is observed in HCEC-1CT cells treated with sEVs from CD patients. This effect is particularly pronounced when cells are exposed to vesicles derived from patients who, after a period of disease control, experience progression (*p* < 0.05 between TTP and CTR, Figure 5I for VIM). Similarly, FLOT-1 gene expression is notably upregulated when cells are treated with sEVs from patients at T3, showing a significant increase compared to CTR (*p* < 0.05), and one compared to T0 (*p* < 0.05). However, a downregulation of FLOT-1 expression is observed at T9 and TTP compared to T3 (*p* < 0.005 and *p* < 0.05, respectively), although its levels remain higher than those observed in untreated control cells (Figure 5J).

The expression levels of the same genes were analysed in HCEC-1CT cells following a 48 h treatment with sEVs derived from RP patients. Notably, no significant changes in expression were observed for PDGFRB and PPARG during the treatment period (Figure 5K,L), neither for PGP, VEGFA, nor for CDH1 (Figure 5 M,O,R, respectively). In contrast, a significant upregulation was detected in ANGPT2, an increased expression being observed both at T0 versus the control (CTR) and T3 versus the control (*p* < 0.05) (Figure 5N). Similarly, COL1A1 expression was significantly elevated at both T0 and T3 when compared to the control (both *p* < 0.05) (Figure 5P). An unexpected result was the decrease in CDH2 after treatment with sEVs at both times considered for RP patients compared to control cells (Figure 5R). Furthermore, a statistically significant increase was evident in VIM expression, more specifically in the comparison between T3 and T0 (*p* < 0.05) (Figure 5S). A striking upregulation was also observed for FLOT-1, where both treatment time points (T0 and T3) exhibited significantly higher expression levels compared to the control (*p* < 0.05) (Figure 5T). These results suggest that sEVs derived from RP patients may induce modifications in the ECM and promote EMT processes. This could have significant implications on disease progression or cellular adaptation mechanisms in response to signals present in the microenvironment.

**Figure 6:**
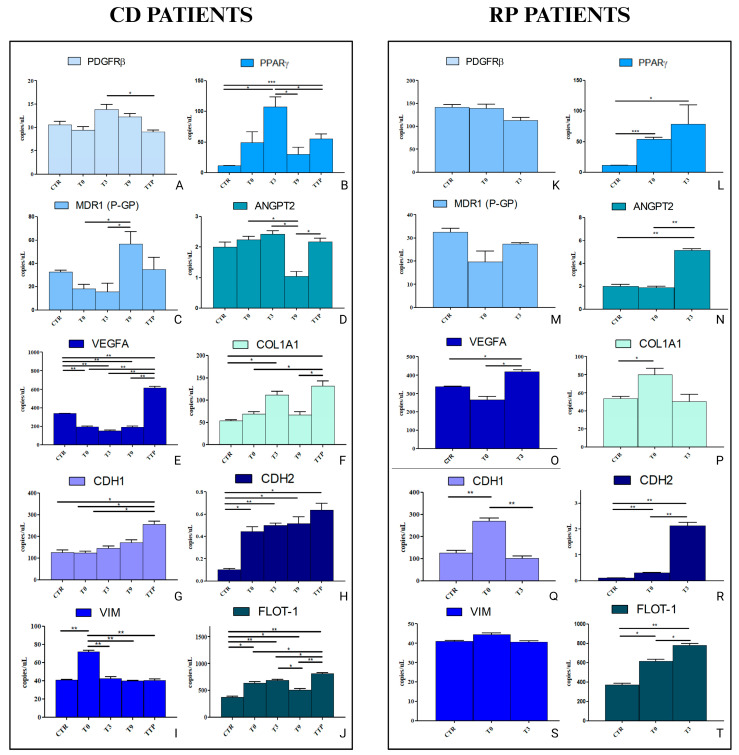
Droplet digital PCR analysis of PDGFRB, PPARG, MDR1 (PGP), ANGPT2, VEGFA, COL1A1, CDH1 (E-CAD), CDH2 (N-CAD), VIM, and FLOT-1 in the HEPA-RG cell line treated with serum-derived sEVs from patients with GC, Controlled Disease (CD), and Rapid progression (RP) at T0 (before the first cycle of PTX) before the third cycle (T3), before the ninth cycle (T9) and at progression (TTP), for CD, and at T0 and T3 for RP patients. The value of copies/μL for PDGFRB is reported in (**A**), PPARG is reported in (**B**), MDR(PGP) is reported in (**C**), ANGPT2 is reported in (**D**),VEGFA is reported in (**E**), COL1A1 is reported in (**F**), CDH1 (E-CAD) is reported in (**G**), CDH2 (N-CAD) is reported in (**H**), VIM is reported in (**I**), and FLOT-1 is reported in (**J**) for CD patients, while the same genes are reported from (**K**) to (**T**), with the same sequence. Statistical analysis was performed using one-way ANOVA with Bonferroni correction for multiple comparisons, conducted in SPSS. Statistical significance was defined as follows: * *p* < 0.05; ** *p* < 0.005; *** *p* < 0.0005.

The same experiments were repeated in a liver cell line, HEPA-RG cells, to investigate in vitro the effects of sEVs at an elective site of GC metastasis (Figure 6). Similarly to the HCEC-1CT cells, the HEPA-RG cells were treated with sEVs derived from both CD and RP patients at various time points: T0, T3, T9, and TTP for CD patients, and T0 and T3 for RP patients. Surprisingly, PDGFRβ did not exhibit an upregulation in expression. On the contrary, following treatment with T3, a significant reduction in expression was observed upon stimulation with TTP. This finding is particularly relevant given the well-established role of PDGFRβ in tumour progression and stromal interactions, suggesting that the hepatic microenvironment may respond differently to external stimuli (Figure 6A). Conversely, PPARγ demonstrated a significant increase in expression when cells were treated with vesicles from T3 (*p* < 0.05) and TTP (*p* < 0.0005) compared to the control. However, compared to T3, a significant reduction in expression was observed at T9 (*p* < 0.05) and TTP (*p* < 0.05), although TTP still exhibited higher expression levels compared to T9. Given the known involvement of PPARγ in lipid metabolism and differentiation, this modulation may indicate a shift in cellular phenotype upon prolonged exposure to tumor-derived sEVs (Figure 6B). Regarding MDR1, its expression was markedly upregulated at T9 compared to both T0 and T3, though it did not differ significantly from the time of progression (TTP) in these patients. The overexpression of PGP, a key efflux pump associated with drug resistance, underscores the potential involvement of sEVs in conferring a more resistant phenotype over time (Figure 6C). ANGPT2 displayed a significant reduction in expression when cells were treated with vesicles from T9, compared to T0, T3, and TTP (*p* < 0.05). This suggests that the angiogenic response may be transiently suppressed at this stage, potentially affecting endothelial remodelling in the hepatic microenvironment (Figure 6D). Notably, VEGFA expression was reduced at T0, T3, and T9 compared to the control (*p* < 0.005), whereas at TTP, its expression significantly increased compared to all conditions and the control (*p* < 0.005) (Figure 6E). This pattern suggests a delayed but strong pro-angiogenic shift in the later stages of disease progression, likely contributing to an enhanced metastatic niche. COL1A1, a key ECM component, exhibited higher expression levels compared to the control, particularly at T3 and TTP (*p* < 0.05), and TTP also showed increased expression compared to both T0 and T9 (*p* < 0.05). The persistent upregulation of COL1A1 reinforces the idea of an evolving fibrotic and pro-migratory microenvironment in response to tumour-derived signals (Figure 6F). Unlike what was observed in HCEC-1CT cells, CDH1 (E-CAD) expression increased significantly when HEPA-RG cells were treated with sEVs from TTP patients, both compared to the control and to T0 and T3 (*p* < 0.05). In contrast, no significant differences were observed at other time points compared to the control (Figure 6G). This finding suggests that, in hepatic cells, the late-stage metastatic process might not strictly follow a classical EMT pattern but could instead involve a partial EMT or hybrid states where epithelial features are maintained alongside mesenchymal characteristics. On the other hand, CDH2 (N-CAD) showed a consistent increase in expression at all time points compared to the control (*p* < 0.05 and *p* < 0.005), indicating a persistent mesenchymal-like shift (Figure 6H). The concurrent upregulation of both E-cadherin and N-cadherin suggests a dynamic EMT process, in which cells acquire mesenchymal properties while retaining some epithelial traits, a phenomenon increasingly recognized in metastatic progression. Overall, these findings underscore the complexity of EMT dynamics in hepatic metastasis. The differential regulation of key EMT markers and ECM components suggests that sEVs play a pivotal role in orchestrating a permissive microenvironment for metastatic colonization. The observed upregulation of mesenchymal markers (CDH2, COL1A1), alongside the late stage increase in VEGFA (TTP Vs T0 with a *p* < 0.05), points to a progressively evolving metastatic niche characterized by ECM remodelling, angiogenesis, and potential resistance mechanisms (Figure 6I). A significant increase of gene expression was noticed for FLOT-1 in all time point conditions compared to the CTR and between TTP and the other points (*p* < 0.05 and *p* < 0.005, respectively) (Figure 6J). The analysis of gene expression in RP patients highlights key mechanisms related to resistance, angiogenesis, and tissue remodelling. PDGFRβ expression remains stable across the tested conditions, indicating its consistent presence in RP patients (Figure 6K). PPARγ, on the other hand, shows a significant increase at T0 compared to the control (*p* < 0.0005) and remains elevated at T3 (Figure 6L). This suggests a crucial role in both the metabolic and inflammatory regulation in RP. Interestingly, MDR1 does not exhibit significant variations across conditions, hinting at an initial stability of this gene in these stimulated cells (Figure 6M). In contrast, ANGPT2 expression rises significantly at T3 compared to T0 and the control (*p* < 0.005), pointing to its involvement in angiogenesis regulation and vascular permeability (Figure 6N). This effect is further supported by VEGFA, which also shows significant variations between T0 and T3, as well as between the control and T3 (*p* < 0.05), reinforcing the critical role mediated by sEVs in vascularization within RP patients (Figure 6O). Regarding ECM remodelling, COL1A1 expression increases at T0 compared to the control (*p* < 0.05), suggesting its potential involvement in fibrosis and structural tissue changes (Figure 6P). Additionally, the expression patterns of CDH1 and CDH2 suggest a role in the EMT, an important process disease progression. In particular, CDH1 is significantly upregulated at T0 compared to both the control and T3, while CDH2 increases at both T0 and T3 compared to the control and also between T3 and T0 (*p* < 0.005) (Figure 6Q,R). This suggests a dynamic regulation of cell adhesion molecules, possibly influencing EMT-related pathways. Interestingly, VIM does not show significant expression changes (Figure 6S), indicating early activation of the mesenchymal process mediated by CDH1 and CDH2. However, the progressive increase in FLOT-1 expression in T0 versus CTR with *p* < 0.05, in T3 versus CTR with *p* < 0.005, and in T3 versus T0 with *p* < 0.05 points to a potential role in lipid raft-associated signalling and cellular regulation (Figure 6T). Overall, these findings show that RP patients exhibit mechanisms linked to resistance, angiogenesis, and tissue remodelling, and especially EMT and ECM modifications that may contribute to disease progression.

### 3.5. Analysis of Protein Expression in HCEC-1CT and HEPA-RG Cells Stimulated with sEVs from CD and RP Patients with Metastatic Gastric Cancer

**Figure 7:**
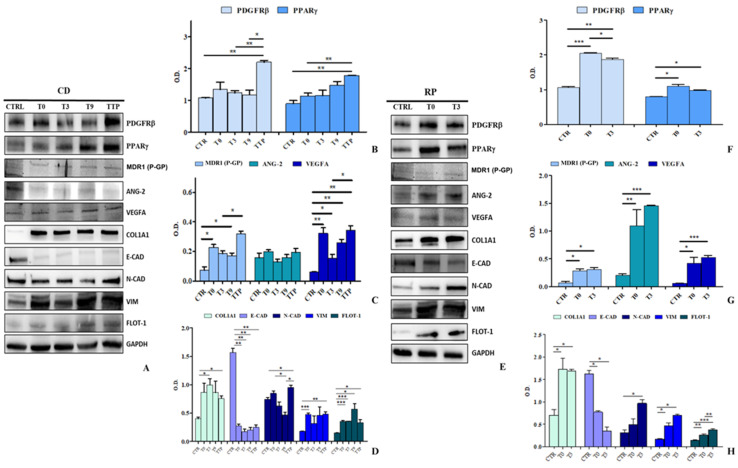
Evaluation of several proteins involved in EMT, ECM and Chemoresistance in the HCEC-1CT cell line treated with serum-derived sEVs from patients with GC, Controlled Disease (CD), and Rapid progression (RP) at T0 (before the first cycle of PTX) before the third cycle (T3), before the ninth cycle (T9) and at progression time (TTP), for CD, and at T0 and T3 for RP patients Representative Western blotting of different proteins (PDGFRβ, PPARγ, MDR1(P-gp), ANG-2, VEGFA, COL1A1, E-CAD, N-CAD, VIM, and FLOT-1) and housekeeping protein (GAPDH) (**A**) after treatment with sEVs. Semiquantitative evaluation of considered proteins expression levels in HCEC-1CT treated with CD sEVs (**B**–**D**). Representative Western blotting of treated cells with sEVs for the same proteins in patients RP (**E**) and semiquantitative evaluation of considered proteins (**F**–**H**). Statistical analysis was performed using one-way ANOVA followed by Bonferroni correction for multiple comparisons, conducted in SPSS. GAPDH was used for normalization of protein expressions. Statistical significance is indicated as follows: * *p* < 0.05; ** *p* < 0.005; *** *p* < 0.0005. The uncropped bolts are shown in Supplementary Materials.

The expression of key proteins involved in angiogenesis, chemoresistance, EMT, and ECM remodeling was analyzed in HCEC-1CT and HEPA-RG cells treated with sEVs from CD and RP patients. Western blot analysis (Figure 7A) revealed a significant upregulation of PDGFRβ in HCEC-1CT cells treated with CD patient-derived sEVs at TTP (*p* < 0.005), compared to control, T3, and T9. PPARγ also showed a noticeable overexpression at T9 and TTP (*p* < 0.05) (Figure 7B). P-gp, ANG-2, and VEGFA were differentially regulated. P-gp was significantly increased at T0 and T9 (*p* < 0.05), with a further elevation at TTP. VEGFA was markedly overexpressed at all time points (*p* < 0.005), showing a progressive upregulation from T0 to TTP. Conversely, ANG-2 levels remained unchanged (Figure 7C). Regarding EMT and ECM markers, COL1A1 was consistently overexpressed across all time points (*p* < 0.05). A drastic E-CAD downregulation indicated EMT activation, while N-CAD initially increased, dropped at T9, and then surged again at TTP, suggesting, in the same way as the E-CAD trend, EMT reactivation (*p* < 0.05). VIM showed dynamic modulation, peaking at T0 (*p* < 0.0005) and TTP (*p* < 0.005), while declining at T3 and T9. Notably, FLOT-1, a key EMT and metastasis-associated protein, was significantly elevated at all time points, particularly at T0 and T3 (*p* < 0.0005). These findings highlight sEVs-driven EMT modulation and reinforce a critical pro-metastatic role for FLOT-1 in tumor progression. The most compelling evidence of the highly pro-metastatic activity is observed when HCEC-1CT cells are stimulated with sEVs derived from patients exhibiting rapid disease progression (Figure 7D). The representative Western blot analyses of the examined markers are presented in Figure 7E. Notably, these patients demonstrate a swift and pronounced evolution in the expression levels of the analysed proteins, further underscoring the aggressive nature of their disease progression. The PDGFRβ have a crucial role in the ANG-2 stimulation, promoting vascular destabilization and aberrant angiogenesis. Furthermore, this enhances the formation of new tumor blood vessels, with an increase of VEGFA expression, thereby increasing invasiveness and metastatic potential. PDGFRβ upregulates P-gp expression, contributing to chemoresistance and the tumour’s protection against therapeutic interventions. Moreover, it plays a pivotal role in EMT and serves as a key regulator of tumour progression. Notably, stimulation of HCEC-1CT cells with RP-derived sEVs led to a significant upregulation of PDGFRβ at both T0 and T3 compared to the control (*p* < 0.0005 and *p* < 0.005, respectively), a trend mirrored in the increased expression of PPARγ (*p* < 0.05) (Figure 7F). Key markers involved in chemoresistance and angiogenesis also exhibited a substantial increase following treatment with RP-derived sEVs (Figure 5G). Specifically, P-gp levels were significantly elevated at T0 and T3 (both *p* < 0.05), while ANG-2 showed a marked rise at both time points (*p* < 0.005). A strong upregulation of VEGFA was observed at T0 (*p* < 0.05) and further amplified at T3 (*p* < 0.0005), underscoring the pro-angiogenic effect of RP derived sEVs (Figure 7G). Furthermore, cells treated with RP-derived sEVs displayed a distinct shift towards ECM remodelling and the EMT. A significant increase in COL1A1 expression was detected at T0 and T3 (*p* < 0.05), while E-CAD expression was notably reduced compared to the control (*p* < 0.05). In contrast, N-CAD and VIM levels were markedly elevated at T0 and T3 (*p* < 0.05), further supporting EMT induction. Importantly, FLOT-1 expression showed a striking increase in cells treated with vesicles from T0 (*p* < 0.005) and T3 (*p* < 0.0005) sEVs, with a significant difference also observed between T0 and T3 (*p* < 0.05), highlighting its potential role in sEV-mediated signaling. These findings provide strong evidence that RP-derived sEVs actively promote tumor aggressiveness, chemoresistance, and angiogenesis, reinforcing their critical role in driving EMT and ECM remodeling in a normal colon cell line (Figure 7H).

**Figure 8:**
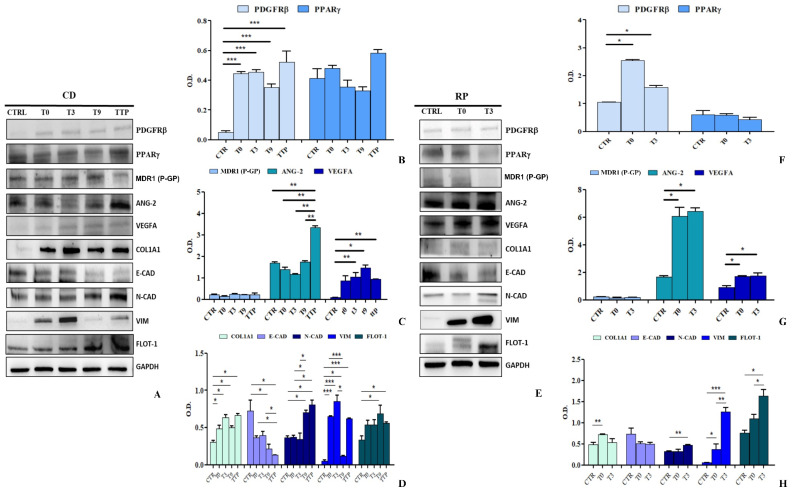
Evaluation of several proteins involved in EMT, ECM and Chemoresistance in HEPA-RG cell line treated with serum-derived sEVs from patients with GC, Controlled Disease (CD), and Rapid Progression (RP) at T0 (before the first cycle of PTX) before the third cycle (T3), before the ninth cycle (T9) and at progression (TTP), for CD, and at T0 and T3 for RP patients Representative Western blotting of different proteins (PDGFRβ, PPARγ, MDR1(P-gp), ANG-2, VEGFA, COL1A1, E-CAD, N-CAD, VIM and FLOT-1) and housekeeping protein (GAPDH) (**A**) after treatment with sEVs. Semiquantitative evaluation of considered proteins expression levels in HCEC-1CT treated with CD sEVs (**B**–**D**). Representative Western blotting of treated cells with sEVs for the same proteins in patients RP (**E**) and semiquantitative evaluation of considered proteins (**F**–**H**). Statistical analysis was performed using one-way ANOVA followed by Bonferroni correction for multiple comparisons, conducted in SPSS. GAPDH was used to normalize protein expression levels. Statistical significance is indicated as follows: * *p* < 0.05; ** *p* < 0.005; *** *p* < 0.0005. The uncropped bolts are shown in Supplementary Materials.

The HEPA-RG cell line was proved to be invaluable for modeling the liver, the primary metastatic site in metastatic gastric cancer. Like HCEC-1CT, these cells were treated for 48 h with sEVs from CD and RP patients. As shown in Figure 8A, representative Western blots display all analyzed proteins. Notably, PDGFRβ expression significantly increased in HEPA-RG cells treated with CD-derived sEVs across all therapeutic time points (*p* < 0.0005), underscoring its crucial role in metastasis. This suggests the activation of pathways involving PDGFRβ signaling as the treatment/time progresses. In contrast, PPARγ levels remained unchanged (Figure 8B). In Figure 8C, the expression of proteins implicated in chemotherapy resistance and angiogenesis was examined in HEPA-RG cells treated with sEVs. Notably, sEVs isolated from these patients did not significantly modulate P-gp expression. In contrast, a pronounced increase in ANG-2 levels was observed in cells treated with sEVs derived from TTP patients, compared with both the control (CTR) and the other chemotherapy treatment time points (TTP vs. CTR and all time points, *p* < 0.005). This figure also illustrates the mean VEGFA expression in cells treated with sEVs obtained from patients at various stages of chemotherapy and at disease progression. A marked elevation in VEGFA was evident at T3 (*p* < 0.005), T9 (*p* < 0.05), and TTP (*p* < 0.005) compared to the control, whereas no significant difference was detected at T0 compared to CTR, indicating a potential involvement in the angiogenic process (Figure 8C). Finally, in Figure 8D, the expression levels of COL1A1 and several EMT-related proteins are illustrated. A significant overexpression of COL1A1 was observed at all time points compared to the control (CTR) (*p <* 0.05), implying a potential matrix remodeling. In contrast, E-CAD levels were significantly reduced when cells were treated with sEVs derived from T9 and TTP samples (*p <* 0.05) relative to CTR. Furthermore, E-CAD also exhibited a significant reduction between T0 and TTP and between T3 and TTP (*p <* 0.05), thus confirming an early activation of EMT. This finding was corroborated by the N-CAD profile, which showed a strong increase in cells treated with sEVs from patients at T9 or at disease progression (TTP) compared to CTR, T0, and T3 (*p <* 0.05). In addition, the figure highlights the trend of VIM expression: compared to CTR, T0 displayed a significant increase, like T3 and TTP (*p <* 0.0005). Moreover, T9 also differed significantly from CTR (*p <* 0.05). FLOT-1, also involved in membrane microdomains and sEVs, is likewise found to be elevated at later time points, T9 versus CTR and TTP versus CTR (*p* < 0.05). Taken together, these data suggest an initial positive response to therapy followed by a subsequent reactivation of the EMT at TTP. The representative Western blotting inherent to HEPA-RG treated with sEVs derived from RP patients is shown in Figure 8E for all evaluated proteins.

In cells treated with sEVs derived from RP patients at both T0 and T3, a significant increase (*p* < 0.05) in PDGFRβ expression was observed compared to the control at both time points, whereas no changes in PPARγ expression was detected (Figure 8F). Similar to PPARγ, P-gp showed no significant modulation compared to the control. In contrast, a significant rise in the expression of ANG-2 and VEGFA was observed in cells treated with T0 and T3-derived sEVs (*p* < 0.05), indicating an enhanced production of angiogenic factors (Figure 8G). Notably, in these cells, unlike those treated with sEVs from CD patients, there was a rapid upregulation of both ECM and EMT related factors, underscoring the tumour aggressiveness mediated by sEVs. In particular, COL1A1 expression was significantly increased in cells exposed to T0-derived sEVs (*p* < 0.05), while N-CAD expression was notably elevated in cells treated with T3-derived sEVs (*p* < 0.005), both compared to the control. Another important EMT marker, VIM, also showed an increased expression in cells treated with patient-derived sEVs at both T0 (*p* < 0.05) and T3 (*p* < 0.0005), as well as between T3 and T0 (*p* < 0.005) compared to the CTR. Moreover, FLOT-1 expression was altered in cells treated with these sEVs, particularly after T3 treatment compared to the control (*p* < 0.05) and also when comparing T3 to T0 (*p* < 0.05) (Figure 8H). Overall, these findings highlight a rapid progression toward a mesenchymal phenotype that is driven by patient-derived sEVs, indicating their fundamental role in promoting tumor aggressiveness. These findings strongly indicate a progressive shift toward a more aggressive/mesenchymal phenotype evidenced particularly by the increase in N-CAD and VIM at the expense of E-CAD, as well as the activation of alternative angiogenesis patterns (PDGF, ANG-2, VEGFA) with advancing treatment. All uncropped Western blot images are provided in Figure S1 of the Supplementary Materials.

## 4. Discussion

GC is among the most prevalent gastrointestinal malignancies worldwide. Its progression to the metastatic stage is driven by multiple factors that converge to foster neoplastic advancement [30]. The development of drug resistance is a major cause of treatment failure and combination regimens address this limitation. Resistance to the taxane PTX is prominent in the treatment of GC and is the result of multiple molecular mechanisms activated by the tumor and its microenvironment [5]. The combination of paclitaxel with the anti-angiogenic agent Ramucirumab in second-line metastatic GC has undoubtedly been a milestone in later-line treatment [16]. However, the therapy’s outcome is influenced by several factors, and, in some cases, treatment becomes ineffective after only a few cycles [13,31]. The hallmarks of PTX-mediated resistance include alterations in drug transporters, such as the upregulation of P-glycoprotein (P-gp), which facilitates drug efflux and consequently diminishes intracellular accumulation and efficacy [13,32]. As previously reported, the characterisation of two PTX-resistant GC cell lines showed an induction of P-gp expression compared to their sensitive counterparts. Furthermore, sEVs derived from the resistant cells had a higher P-gp content than sEVs derived from the sensitive cells. This finding suggests that vesicle trafficking is one-way; resistant tumor cells propagate the tumor’s ability to resist chemotherapy [13,32]. Recent studies highlighted that multidrug resistance (MDR) mechanisms in GC are strongly associated with overexpression of ATP-binding cassette (ABC) transporters, including P-gp, MRP1, and BCRP. These proteins, reducing intracellular drug concentrations, contributed to chemoresistance. Overexpression of MDR proteins has been reported for other chemotherapeutic drugs, including 5-fluorouracil (5-Fu) and doxorubicin (DOX) [33,34]. Targeting these transporters with specific inhibitors or RNA-based therapies has emerged as a promising strategy to reverse MDR in GC.

In addition, structural changes and dysregulation of intracellular signaling pathways, variations in the levels of molecules that regulate the EMT such as E-CAD, N-CAD, VIM, and FLOT-1 [35,36], and modulation of COL1A1 belonging to the ECM further contribute to the development of PTX resistance [37]. A pivotal role in the development of PTX resistance by accelerating the EMT and modulating the ECM is played by sEVs. Through fusion with recipient cells, these vesicles facilitate the transfer and acquisition of new functional attributes, not only within the tumor cells themselves but also in cells located far from the primary tumor site. Consequently, cells at potential metastatic destinations, such as hepatocytes in the context of GC, initially exhibit signs of cellular distress upon receiving sEVs, eventually progressing toward a mesenchymal phenotype [38,39]. Emerging evidence demonstrates that sEVs carry non-coding RNAs such as circRNAs that regulate EMT progression and ECM remodeling in GC. CircRNAs have been implicated in promoting chemoresistance by modulating apoptosis pathways and enhancing cell migration and invasion [40]. Their unique structural stability makes them potential biomarkers for predicting therapeutic response and disease progression in GC patients undergoing PTX treatment.

One of the most striking findings of this study is the pronounced protein overexpression of PDGFRβ following sEVs treatment and in the sEVs derived from GC patients. High PDGF levels may also be linked to resistance mechanisms that trigger alternative pathways that stimulate angiogenesis following therapy that blocks the main VEGF/VEGFR2 pathway [19]. The overexpression of PDGFRβ in metastatic lesions is frequently associated with an unfavourable prognosis, making this factor a potential biomarker for patient stratification [41]. This observation underscores the receptor’s central and regulatory role not only in angiogenesis but also in orchestrating both the EMT and ECM processes. Recent findings suggest that PDGFRβ hyperactivation may also interact with Src-mediated signaling pathways to sustain pro-survival mechanisms under therapeutic pressure [42]. This crosstalk highlights PDGFRβ as a critical node for targeting angiogenesis and EMT simultaneously to overcome resistance challenges in advanced GC cases. Furthermore, inhibiting PDGFRβ signaling has shown potential in enhancing the antitumor effects of chemotherapy. Targeted therapies against PDGFRβ could offer new avenues for treating metastatic GC by reducing tumor angiogenesis and improving drug efficacy [43].

The present study provides compelling evidence that sEVs derived from CD and RP patients with metastatic GC play an essential role in modulating the EMT and remodeling of the ECM, and the activity between both groups of patients is different. Through comprehensive analyses integrating bioinformatics, gene expression, protein profiling, and functional assays in normal colon (HCEC-1CT) and hepatic (HEPA-RG) cell models, these data highlight the complexity of sEVs-mediated signaling in fostering PTX resistance and driving aggressive tumor phenotypes. A key finding involves the dynamic interplay between PDGFRβ and PPARγ, which emerged as central regulators of cell survival, metabolism, and drug response [44]. sEVs derived from patients who experienced CD initially appeared to have downregulated PDGFRβ, correlating with an enhanced sensitivity to PTX. Conversely, as disease progressed (TTP in CD or from the outset in RP patients), sEVs showed a pronounced upregulation of PDGFRβ. This shift was often accompanied by alterations in PPARγ, suggesting a coordinated regulatory axis capable of promoting drug resistance and cellular plasticity. Equally notable was the modulation of P-gp, a critical efflux pump known to reduce intracellular drug accumulation. In both CD and RP contexts, sEVs could either maintain low P-gp levels or a markedly elevated expression, influencing chemoresistance. This duality underscores the importance of sEVs in dictating the sensitivity or refractoriness of tumor cells (and by extension, the tumor’s microenvironment) to PTX. Several studies underscore the critical role of PDGFRβ in regulating angiogenesis and establishing a tumor microenvironment that propels the progression of metastatic GC. The hyperactivation of this receptor facilitates neovascularization and enhances the survival of malignant cells, rendering PDGFRβ a highly promising therapeutic target for both inhibiting tumor growth and increasing sensitivity to systemic treatments [45,46]. In the present study, the angiogenesis associated proteins, namely VEGFA and ANG-2, were also significantly affected by sEVs, reflecting the importance of vascular remodelling in tumor growth and metastasis. sEVs from CD patients, particularly at mid-treatment time points, frequently downregulated angiogenic signals, mirroring effective therapeutic intervention. However, the same sEVs populations at later progression stages displayed a pronounced upregulation of these pro-angiogenic factors, reinforcing a vascular network supportive of tumor dissemination. Crucially, our data underscore the intense impact of sEVs on ECM remodeling and the EMT. Particularly, the persistent overexpression of COL1A1, a key ECM component, was observed in cells treated with sEVs from both CD and RP group of patients, albeit with distinct temporal patterns. This finding implies an sEV-driven enhancement of ECM density and stiffness, which can inhibit drug delivery and promote invasive capabilities and corroborate the results found in literature. Furthermore, EMT progression was evidenced by a decreased in E-CAD (CDH1) and a concurrent upregulation of N-CAD (CDH2) and VIM. These markers were especially pronounced in cells exposed to sEVs from patients exhibiting rapid disease progression. Intriguingly, some cells displayed a mixed phenotype co-expressing both epithelial and mesenchymal markers, suggesting that sEVs might induce a spectrum of partial EMT states conducive to metastasis. Another noteworthy mediator, FLOT-1, was significantly modulated by sEVs exposure, giving FLOT-1 a role in the membrane’s microdomain organization and endocytosis. Furthermore, elevated FLOT-1 could potentiate the internalization of signaling molecules and further support EMT processes. Its consistent association with sEVs-driven changes underscores how vesicles may harness membrane trafficking pathways to reprogram recipient cells. Mechanistically, these observations align with bioinformatic analyses highlighting PDGFRβ as an upstream regulator of multiple downstream effectors—PPARγ, VEGFA, ANG-2, VIM, COL1A1, and Cadherins all integral to tumor progression, angiogenesis, and cellular plasticity. By ferrying specific cargoes proteins, transcripts, and potentially non-coding RNAs sEVs from GC patients in various treatment phases orchestrate a multifaceted cellular response, effectively “educating” recipient cells to adopt pro-tumorigenic behaviors. From a clinical perspective, the data suggest that monitoring and characterizing circulating sEVs at distinct therapy time points may offer valuable insights into treatment efficacy and emerging resistance. It should be noted that the sEVs were extracted from GC patients who were exposed to the combined action of two drugs; this represents a limitation in studying resistance mechanisms, as a poor response to therapy cannot be unambiguously attributed to resistance mechanisms caused by one of the two drugs. Nevertheless, our study showed that the sEVs content of patients with a good response to therapy is significantly different from the sEVs’ content of patients with a poor response to therapy. Additionally, certain markers that are differentially expressed between the two patient groups may suggest the activation of resistance mechanisms to PTX, such as P-gp overexpression, or to ramucirumab, as seen with proteins involved in alternative angiogenesis pathways to VEGF/VEGFR2, like the overexpression of angiopoietin 2 or PDGF.

Moreover, while in vitro models (HCEC-1CT and HEPA-RG) provided valuable mechanistic insights, they do not fully replicate the complexity of the in vivo tumor microenvironment, where additional cell–cell interactions, immune components, and stromal influences may modulate treatment response. The heterogeneity of sEVs cargo, even within patient subgroups, underscores the challenge of defining consistent biomarkers without large-scale validation. Furthermore, the isolation of EVs by differential ultracentrifugation alone may co-isolate contaminants such as lipoproteins. Future studies will consider additional purification steps to improve the purity of EVs. Finally, although functional associations have been identified between sEVs content and resistance pathways, direct causal links—particularly those involving circRNAs and specific signaling axes—require further investigation through targeted gene editing or in vivo knockdown studies. Future research should focus on validating these findings in animal models and prospective clinical cohorts to solidify the therapeutic and diagnostic potential of sEVs profiling in GC.

## 5. Conclusions and Future Direction

In conclusion, the present study rigorously demonstrates the multifaceted role of sEVs in metastatic GC progression and resistance mechanisms. The data unequivocally highlighted that sEVs derived from patients with metastatic GC play a critical role in modulating EMT, ECM remodeling, and chemoresistance, with PDGFRβ acting as a central regulator. These findings suggest that PDGFRβ and related proteins could serve as valuable biomarkers for predicting treatment response. Furthermore, this study underscores the necessity for longitudinal tracking of sEVs cargo evolution during therapy. Future investigations should focus on in vivo validation using metastatic GC animal models to better mimic the tumor microenvironment dynamics. It is imperative to examine combinatorial therapies targeting PDGFRβ signaling and ECM remodeling, as well as to explore innovative RNA-based therapeutics. These directions promise unprecedented insights into precision medicine approaches in metastatic GC treatment.

## Figures and Tables

**Table 1:** List of genes with corresponding assay IDs (Bio-Rad).

Gene Symbol	Gene Name	Assay ID (Bio-Rad)
PDGFRB	Platelet Derived Growth Factor Receptor Beta	qHsaCID0013272
PPARG	Peroxisome Proliferator-activated Receptor Gamma	qHsaCID0011718
PGP	PhosphoGlycoProtein	qHsaCED0002291
ANGPT2	Angiopoietin 2	qHsaCID0017615
VEGFA	Vascular Endothelial Growth Factor A	qHsaCED0043454
COL1A1	Collagen type I alpha 1 chain	qHsaCED0043248
CDH1	E-cadherin	qHsaCID0015365
CDH2	N-cadherin	qHsaCID0015189
VIM	Vimentin	qHsaCID0012604
FLOT1	Flotillin 1	qHsaCED0037092

**Table 2:** Patients Group Characteristics.

	Group	Progression-Free Survival (PFS), Months	Overall Survival (OS), Months	Key Features
1	RP (n = 16)	≤3 months (median: 2.68 months)	Median: 6.30 months	Progression at first evaluation
2	CD (n = 25)	>3 months (median: 10.38 months)	Median: 12.47 months	Stable disease or partial response at initial evaluation continued therapy until progression or toxicity

## Data Availability

The data will be provided by the corresponding author upon request.

## Supplementary Materials

The following supporting information can be downloaded at: https://www.mdpi.com/article/10.3390/cancers17081360/s1, Figure S1: Uncropped Western blot images.
