# Supplementary material for: Unravelling Paclitaxel Resistance in Gastric Cancer: The Role of Small Extracellular Vesicles in Epithelial Mesenchymal Transition and Extracellular Matrix Remodelling
Source: Cancers (Basel). 2025 Apr 18;17(8):1360. doi: 10.3390/cancers17081360 (PMC12025963; doi:10.3390/cancers17081360)

Figure 3-A

PDGFRβ

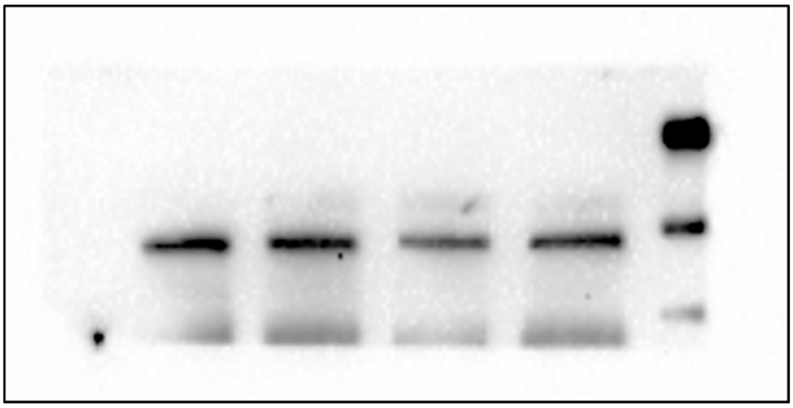

PPARγ

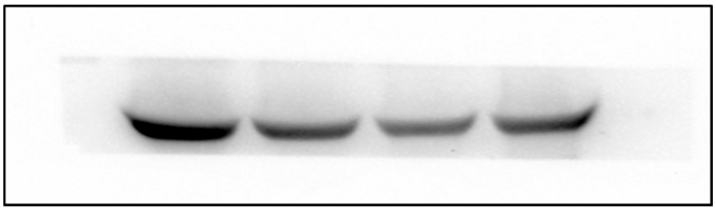

MDR1 (P-GP)

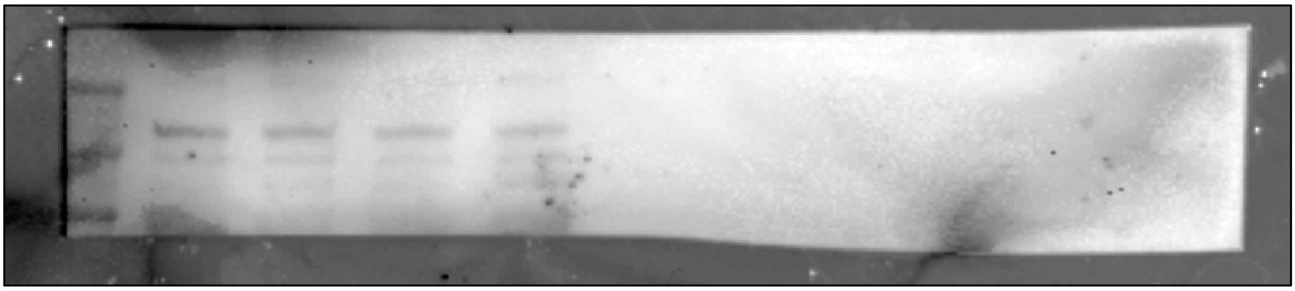

ANG-2

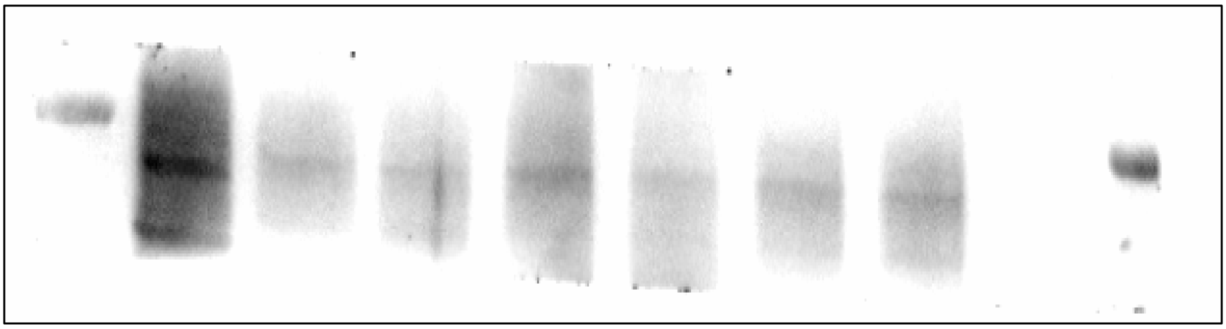

VEGFA

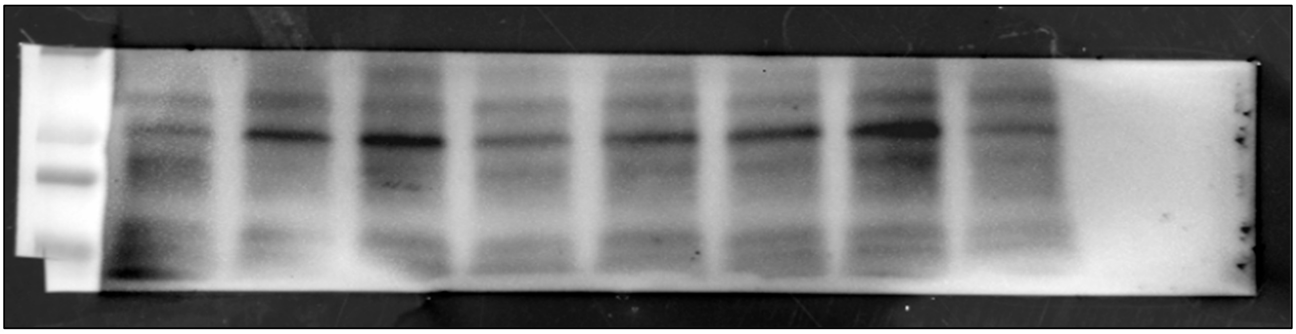

COL1A1

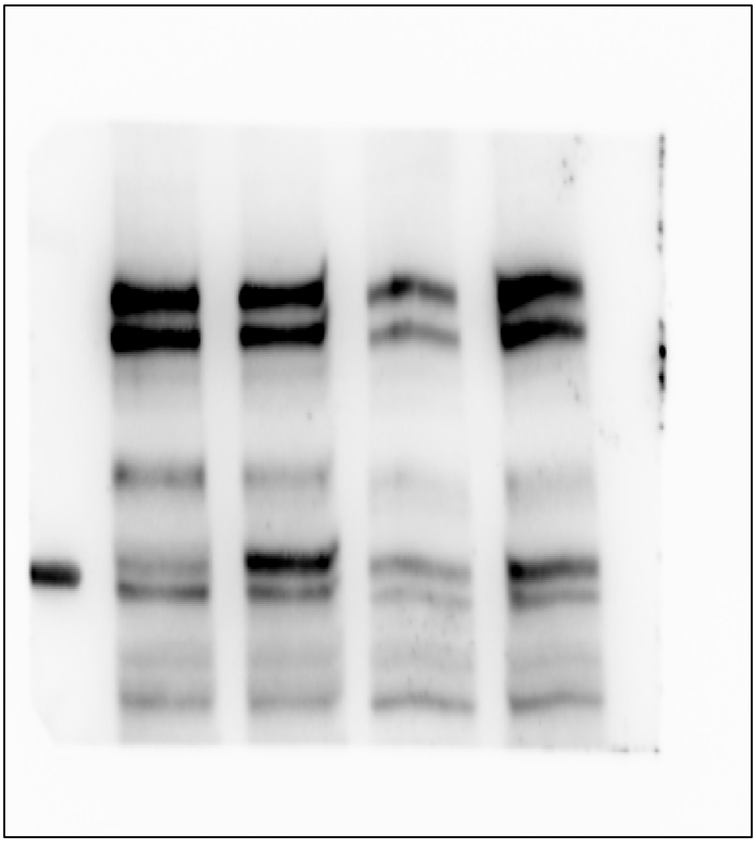

E-CAD

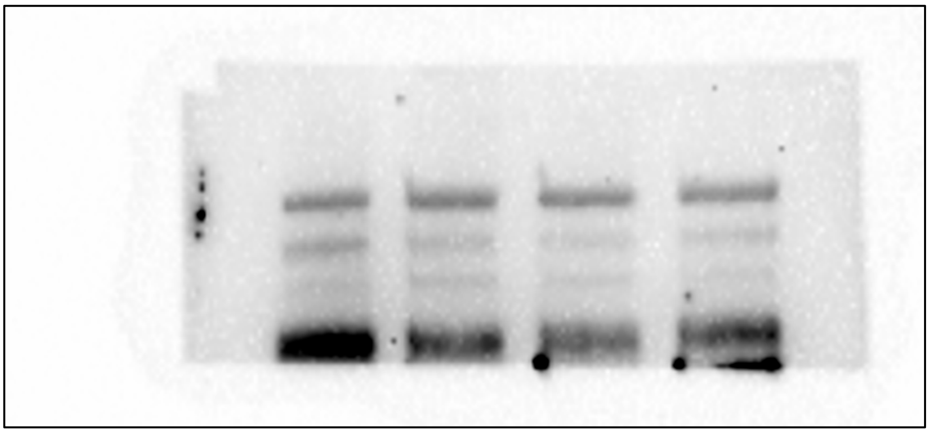

N-CAD

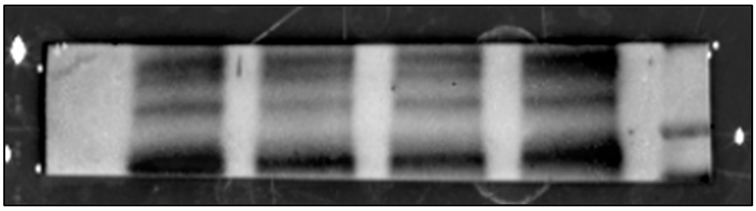

VIM

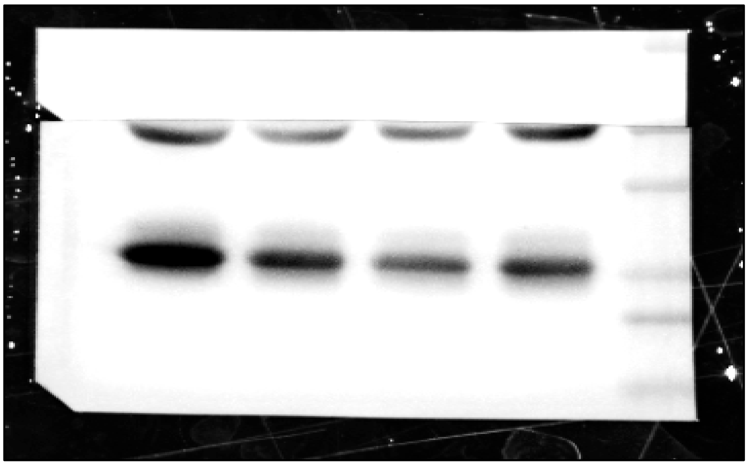

FLOT-1

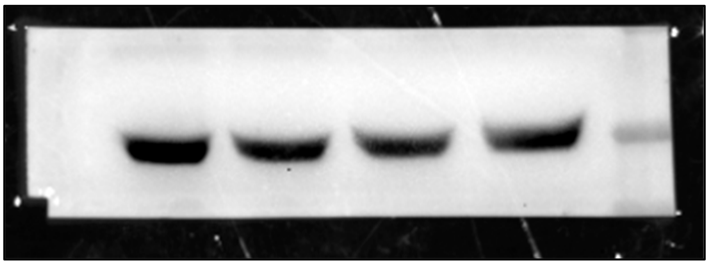

CD63

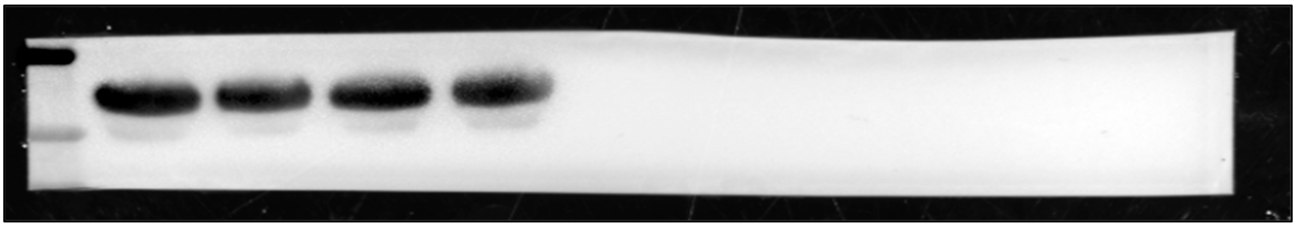

CD81

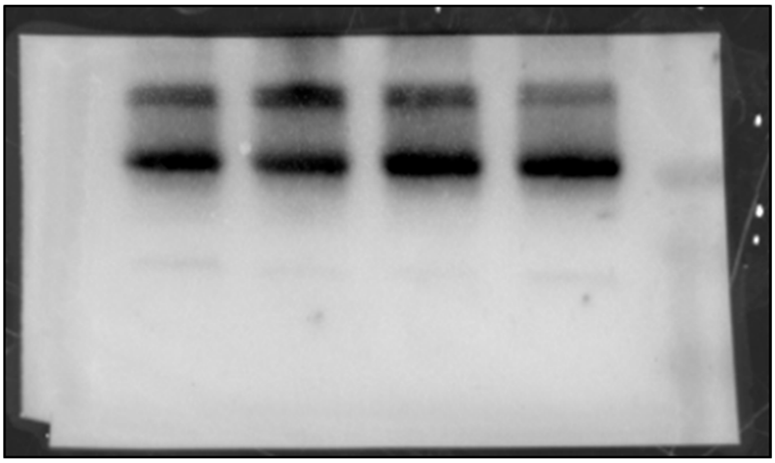

Figure 3-E

PDGFRβ

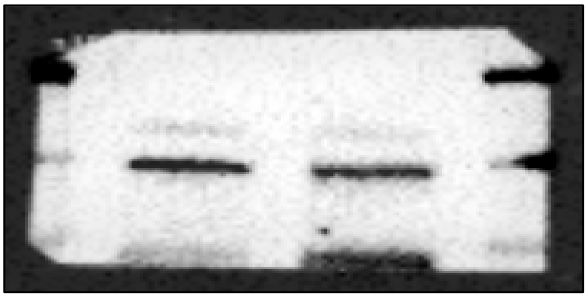

PPARγ

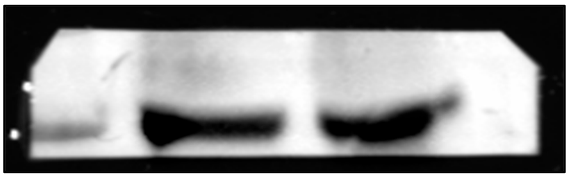

MDR1 (P-GP)

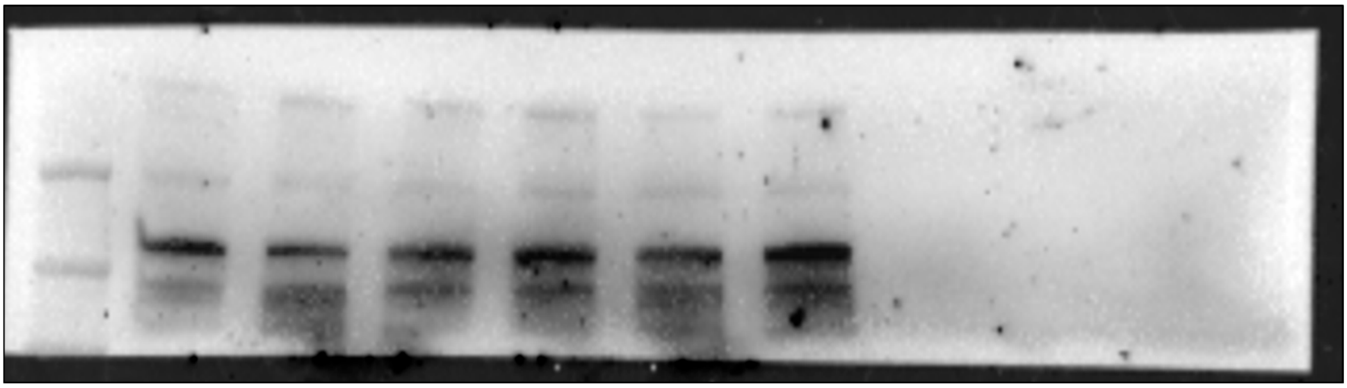

ANG-2

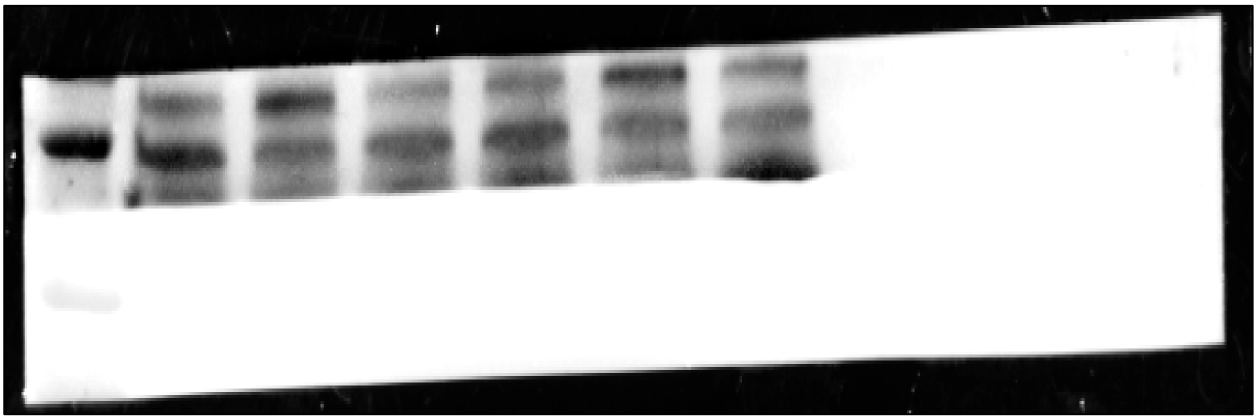

VEGFA

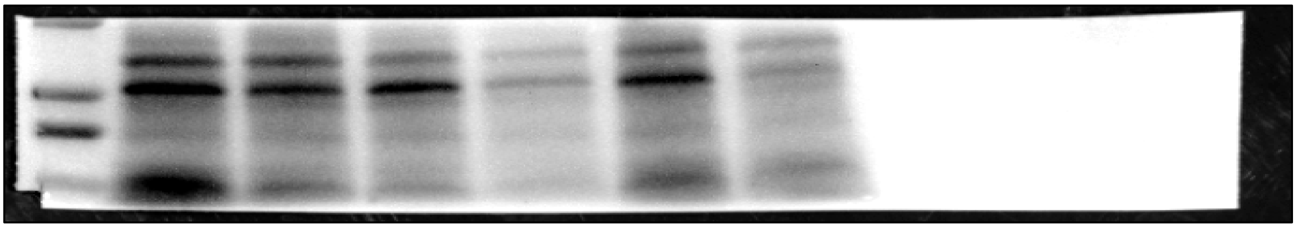

COL1A1

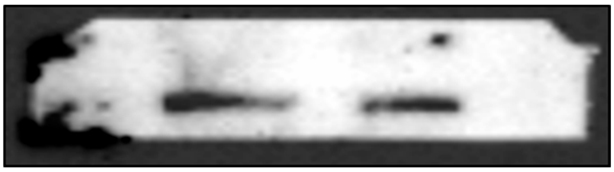

E-CAD

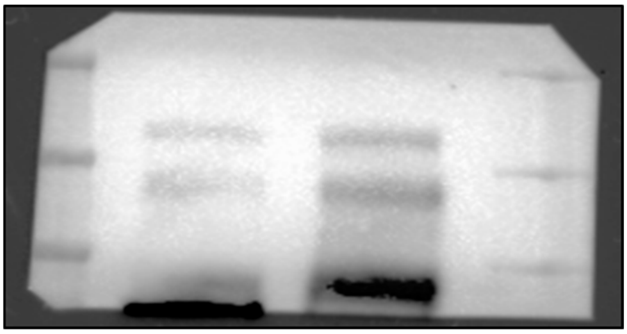

N-CAD

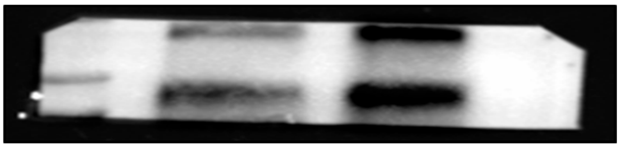

VIM

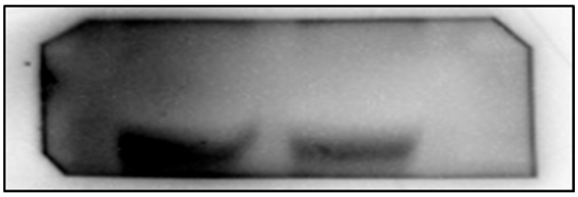

FLOT-1

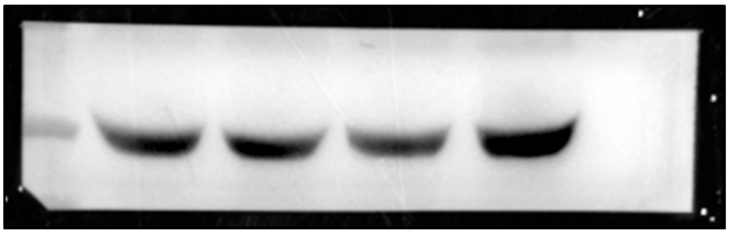

CD63

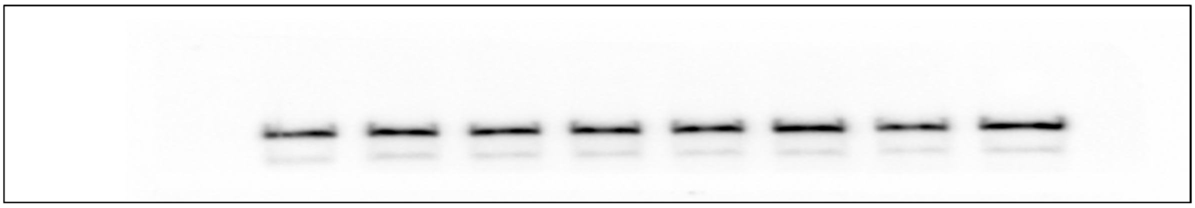

CD81

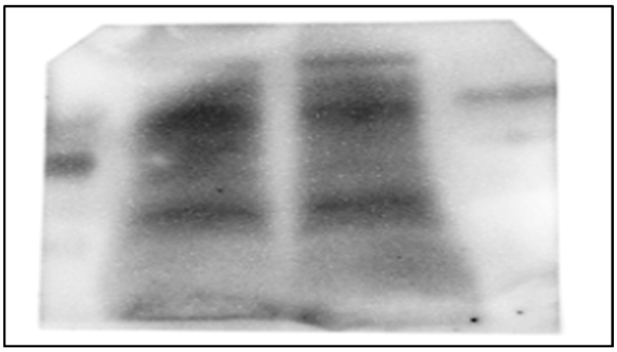

Figure 7-A

PDGFRβ

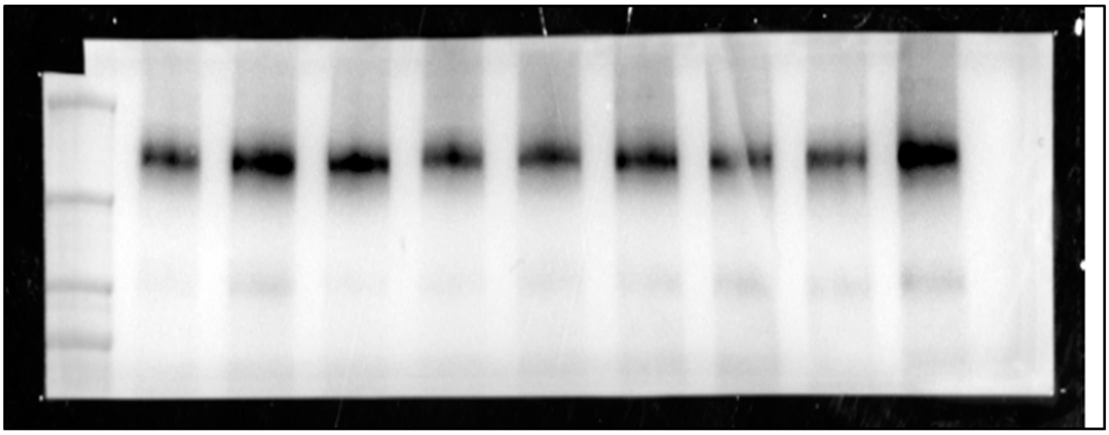

PPARγ

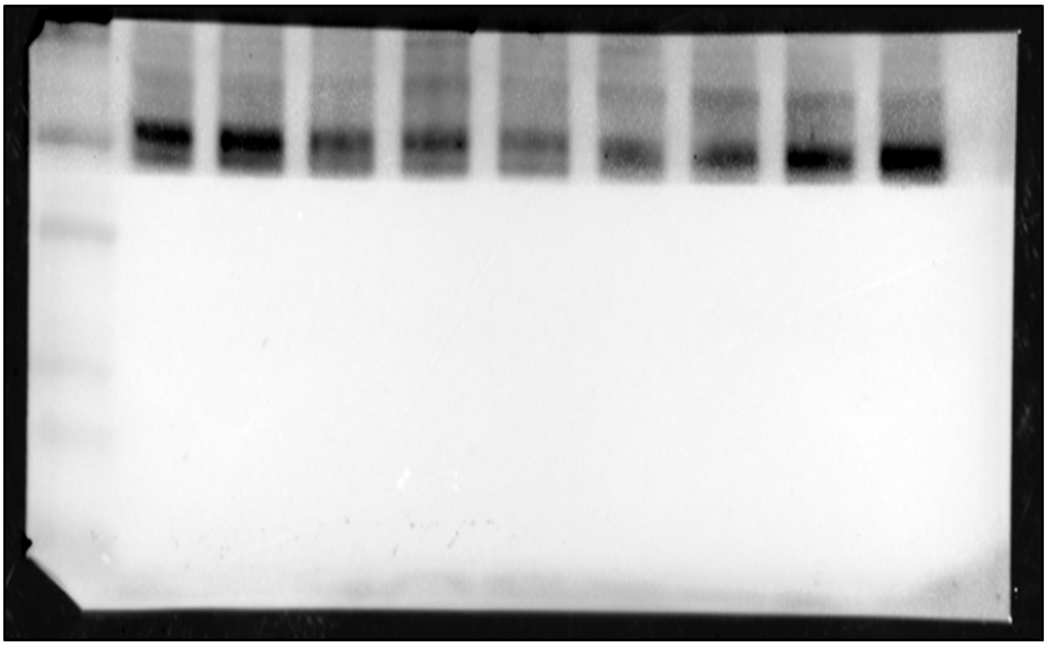

MDR1 (P-GP)

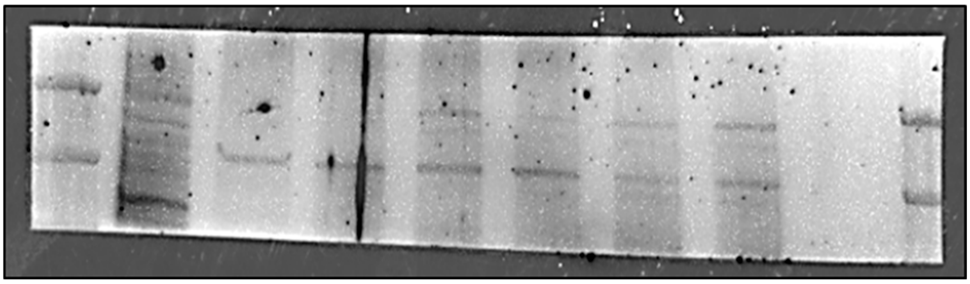

ANG-2

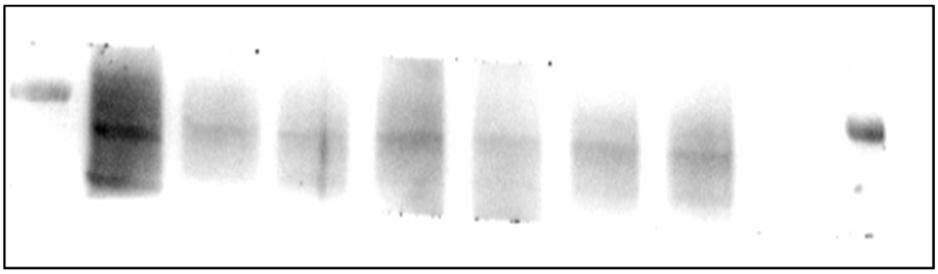

VEGFA

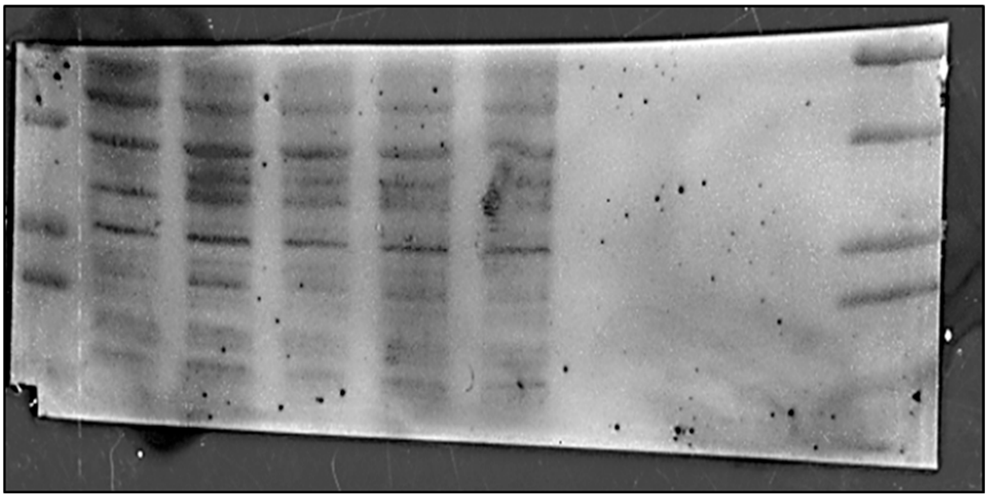

COL1A1

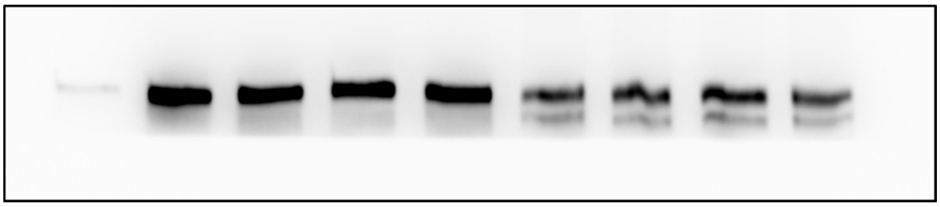

E-CAD

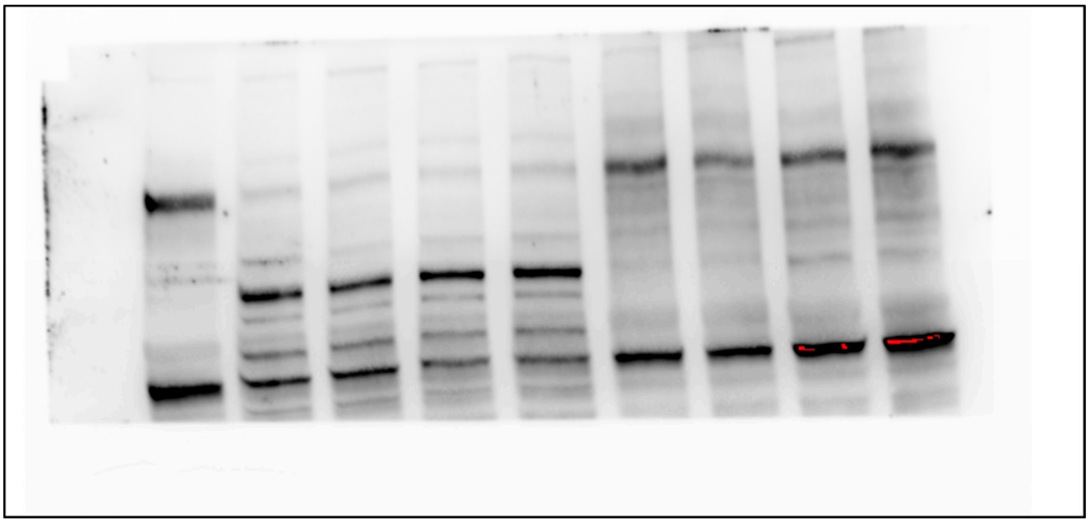

N-CAD

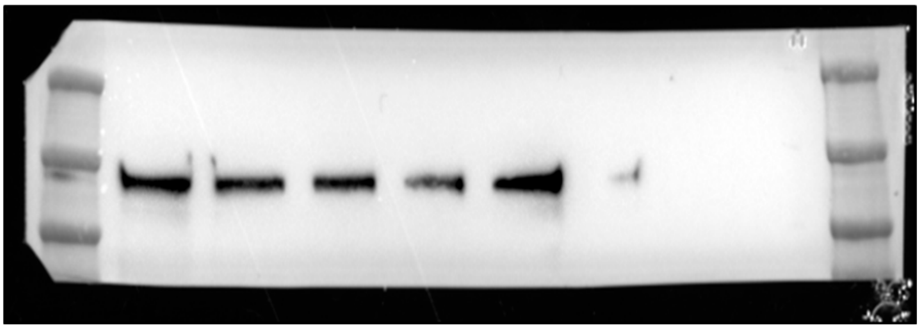

VIM

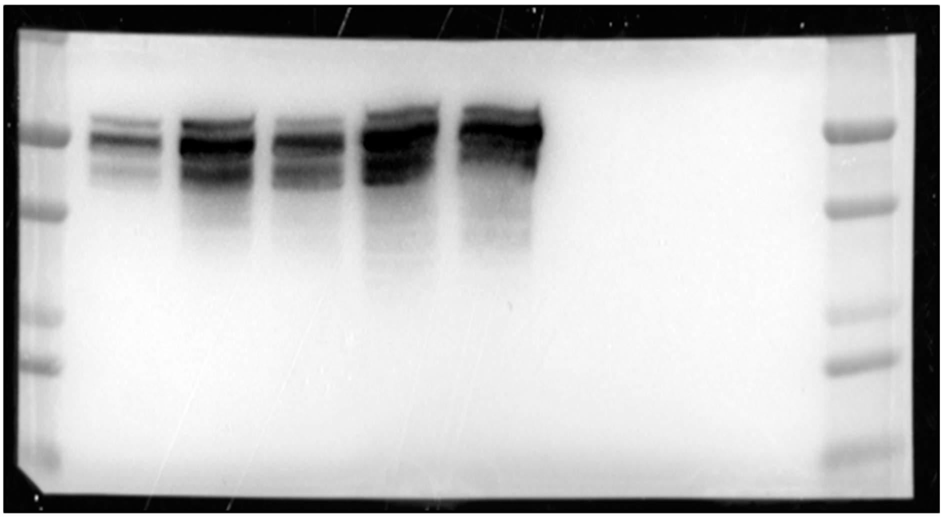

FLOT-1

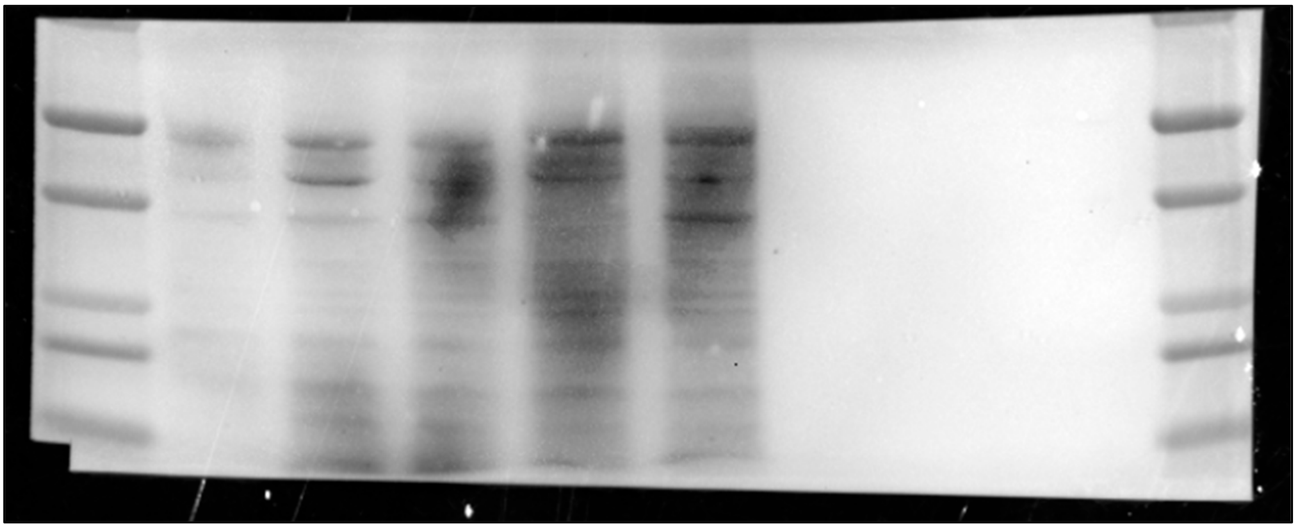

GAPDH

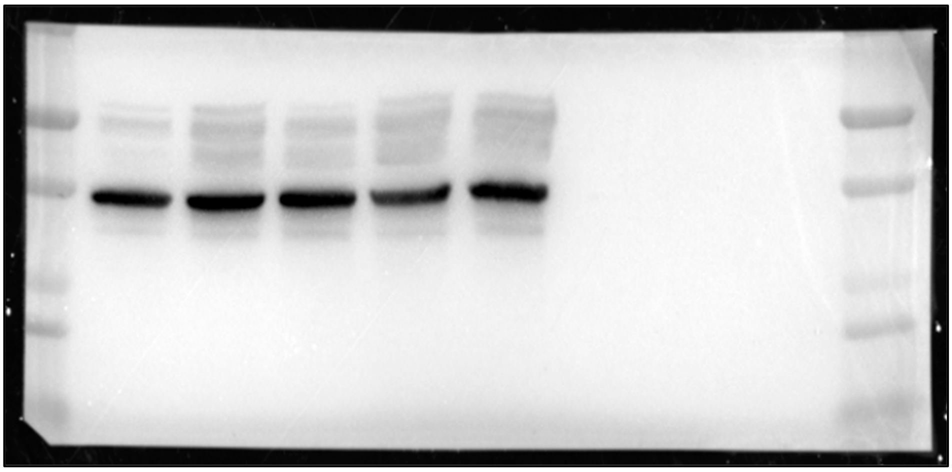

Figure 7-E

PDGFRβ

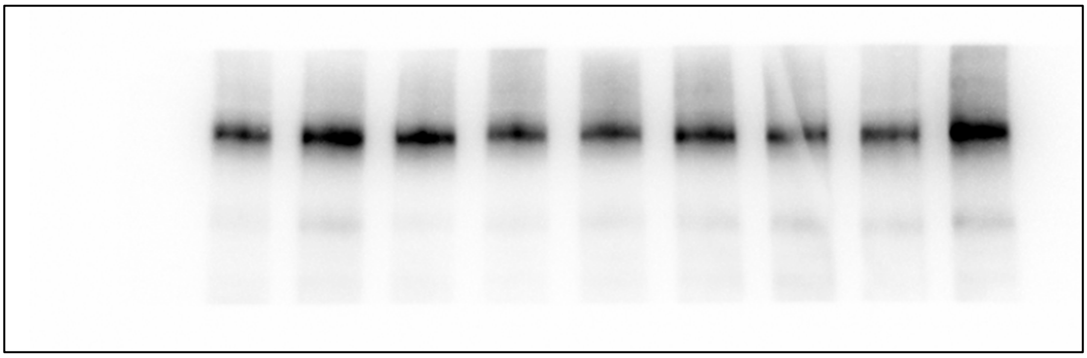

PPARγ

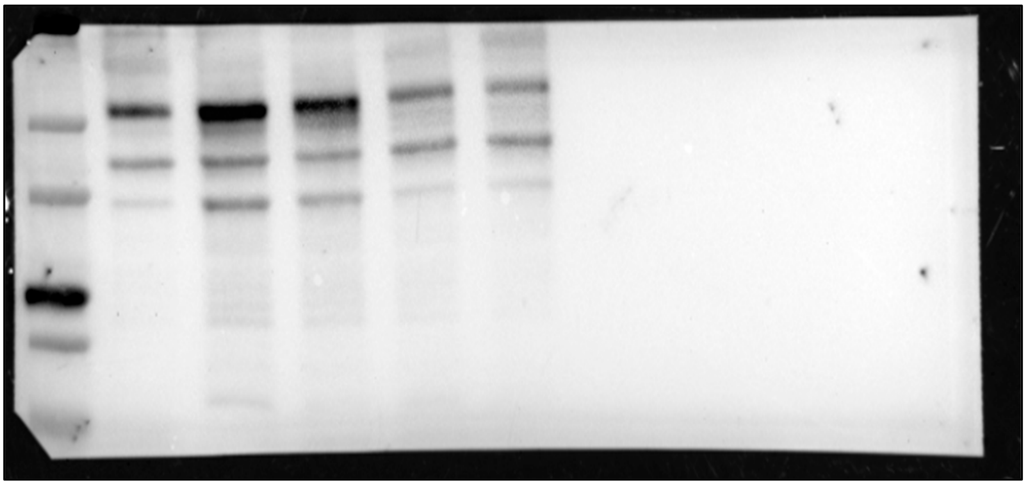

MDR1 (P-GP)

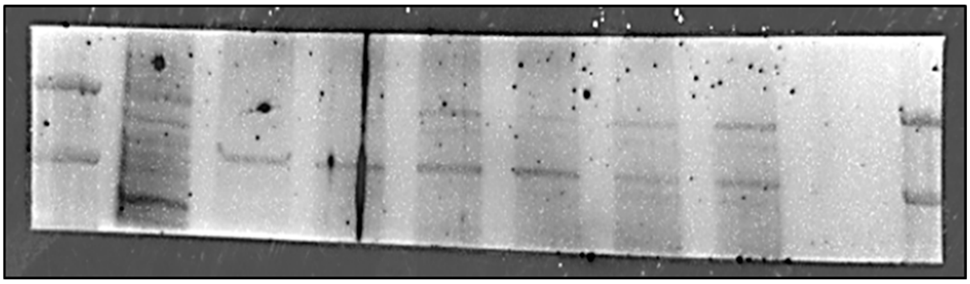

ANG-2

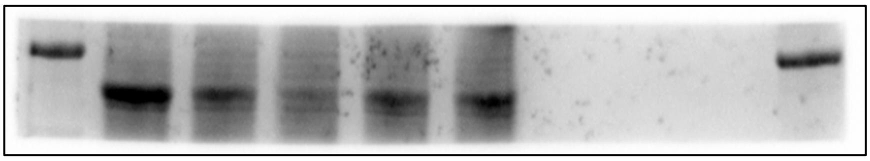

VEGFA

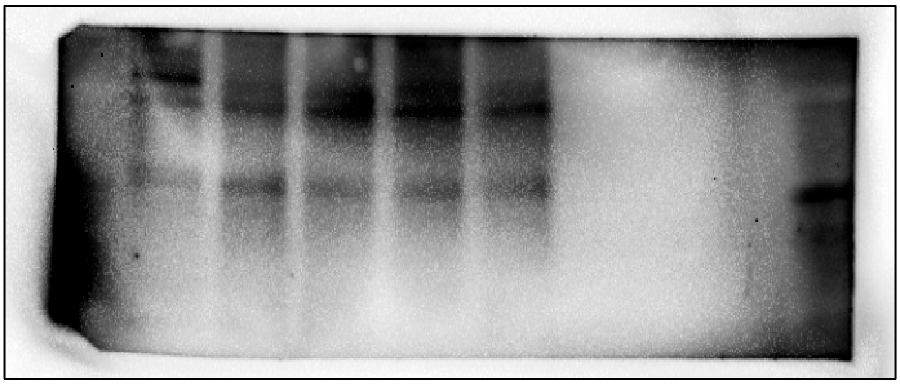

COL1A1

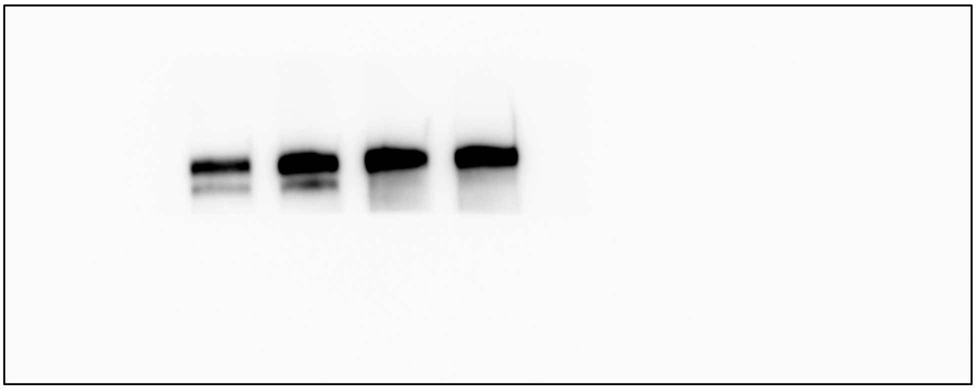

E-CAD

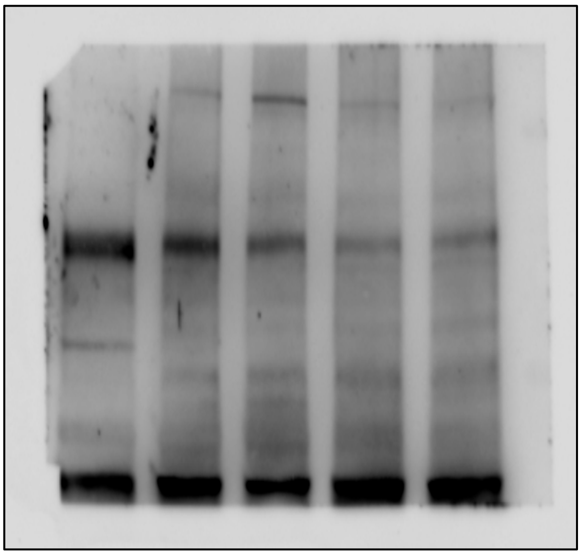

N-CAD

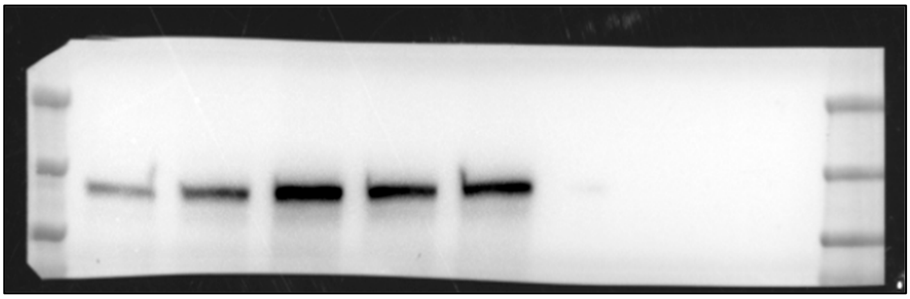

VIM

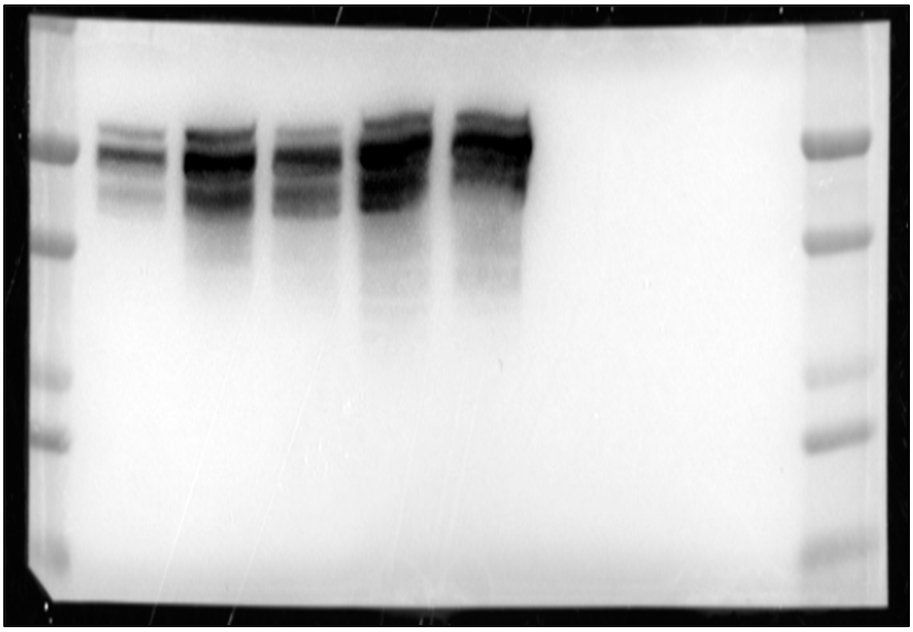

FLOT-1

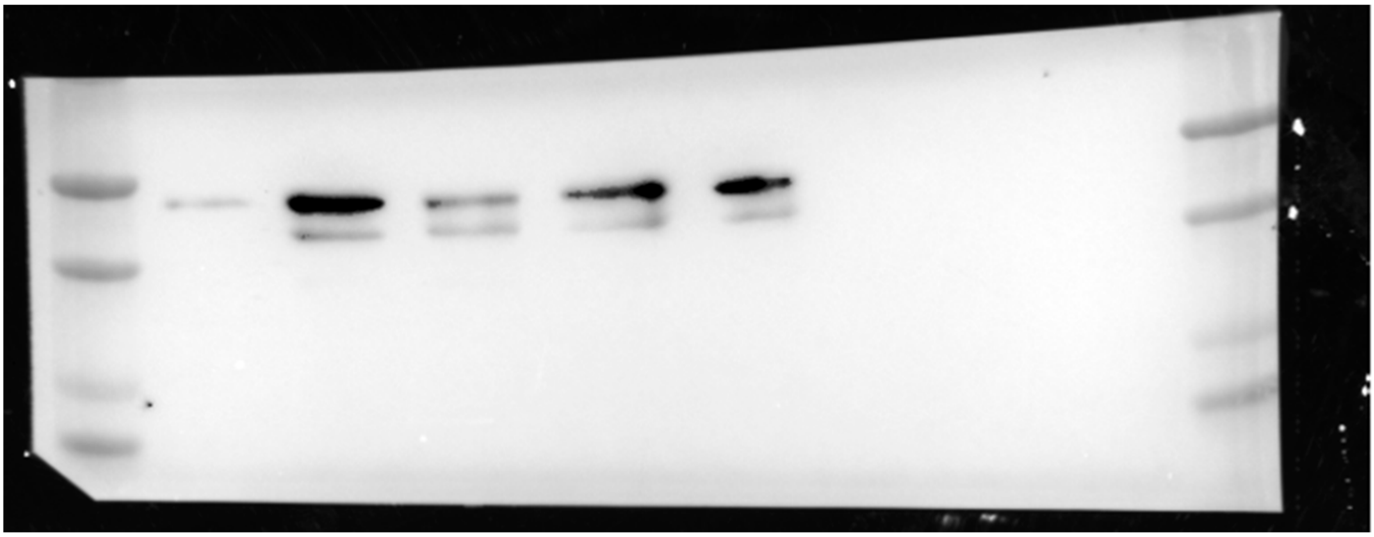

GAPDH

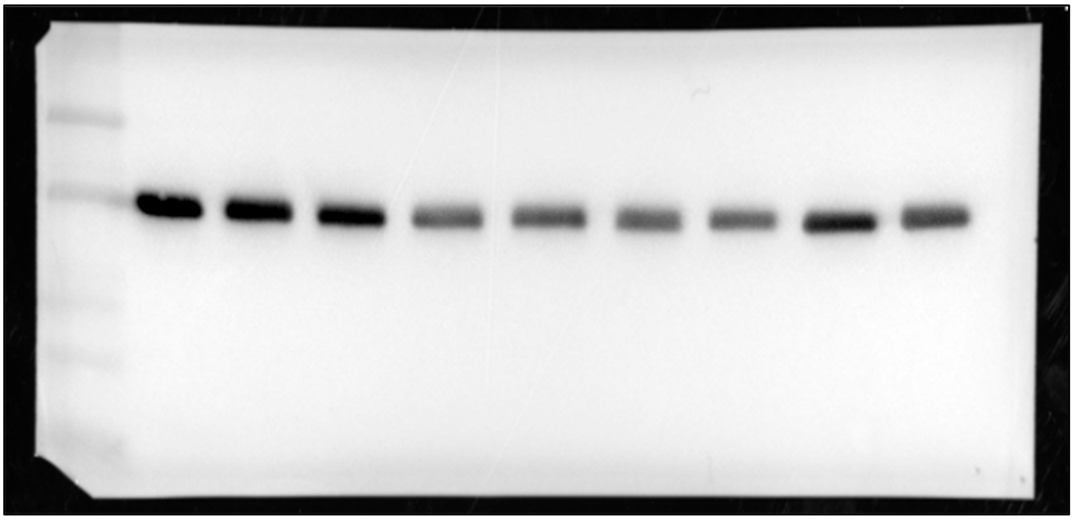

Figure 8-A

PDGFRβ

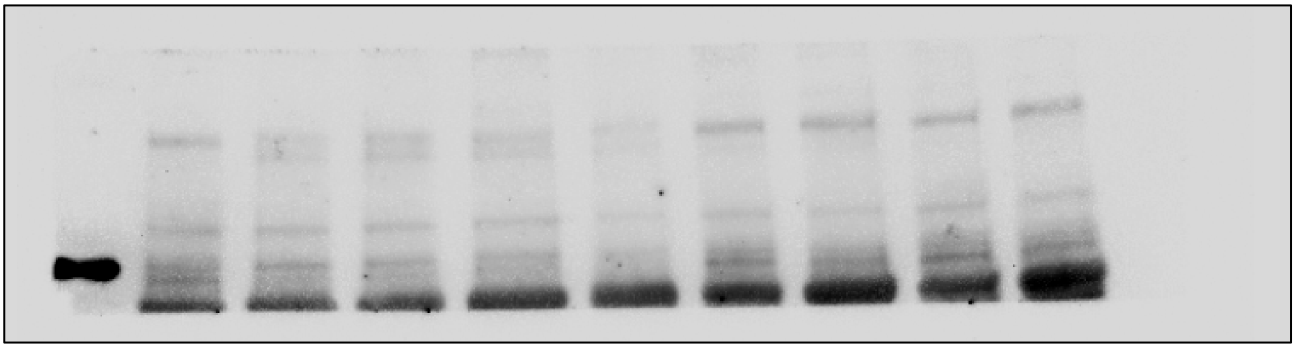

PPARγ

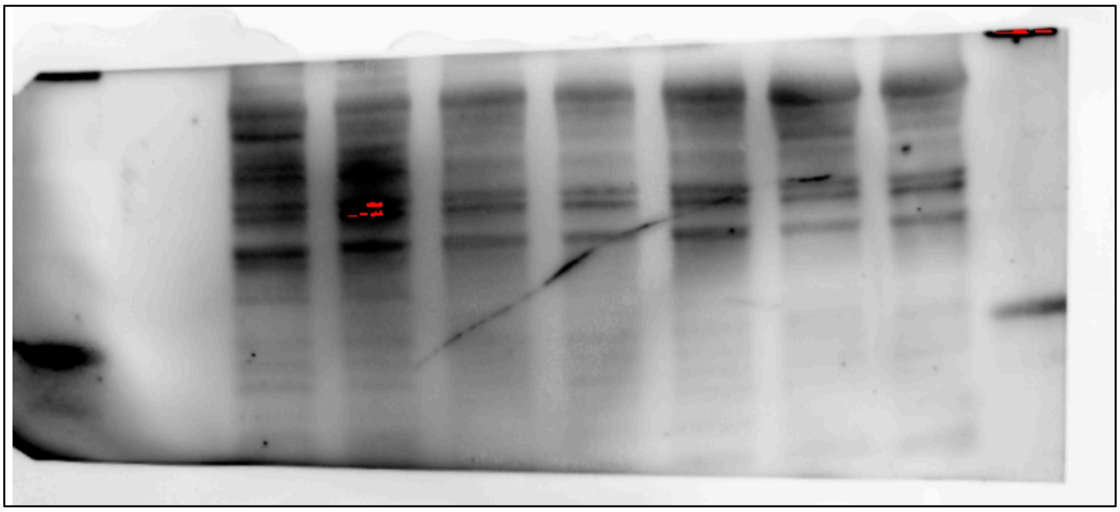

MDR1 (P-GP)

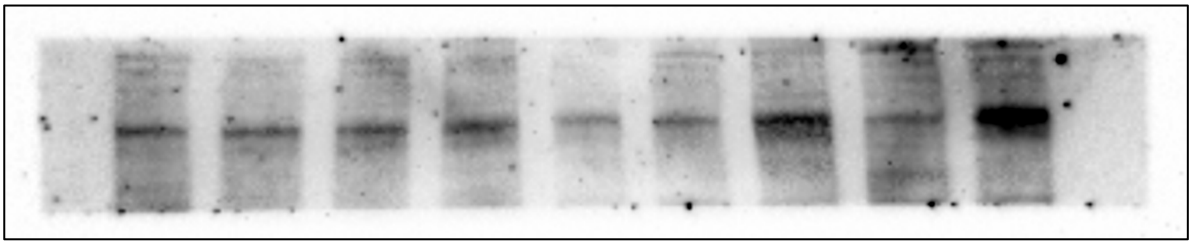

ANG-2

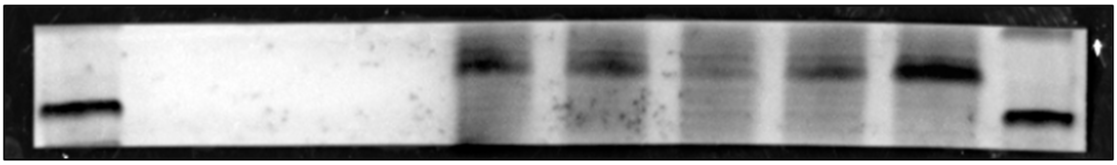

VEGFA

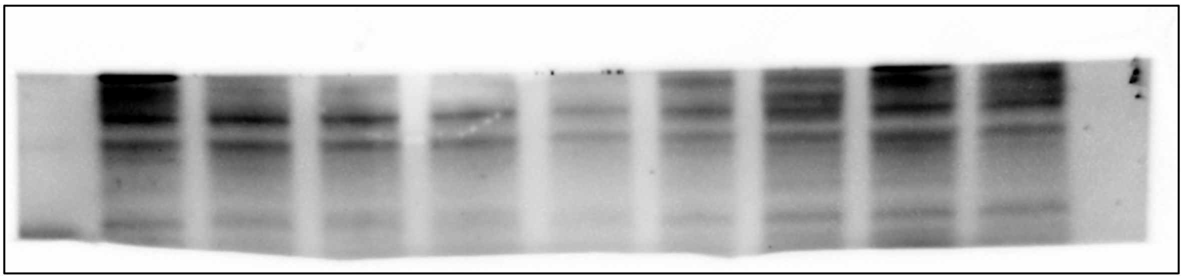

COL1A1

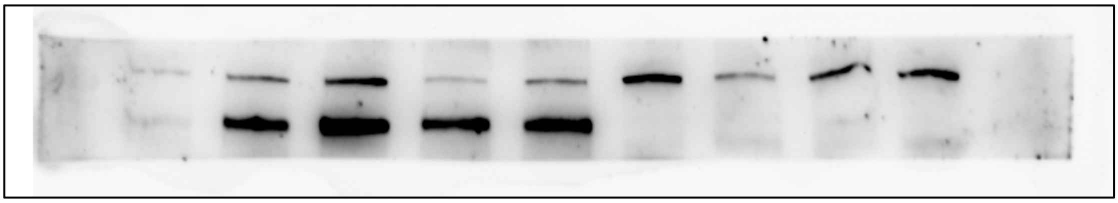

E-CAD

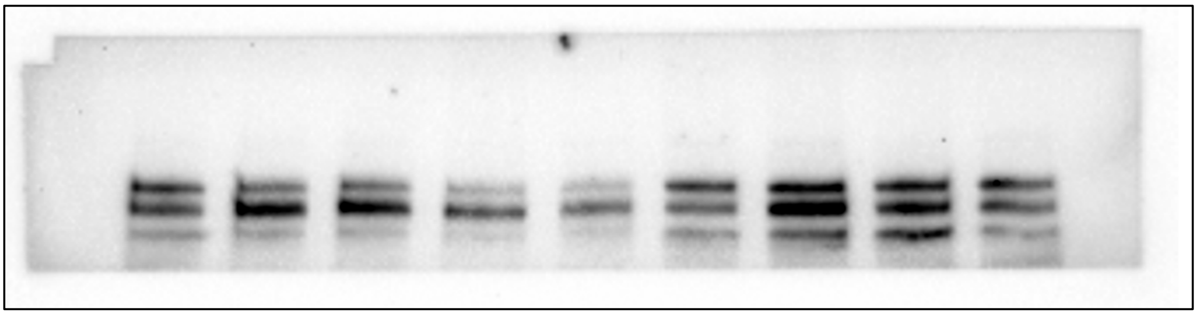

N-CAD

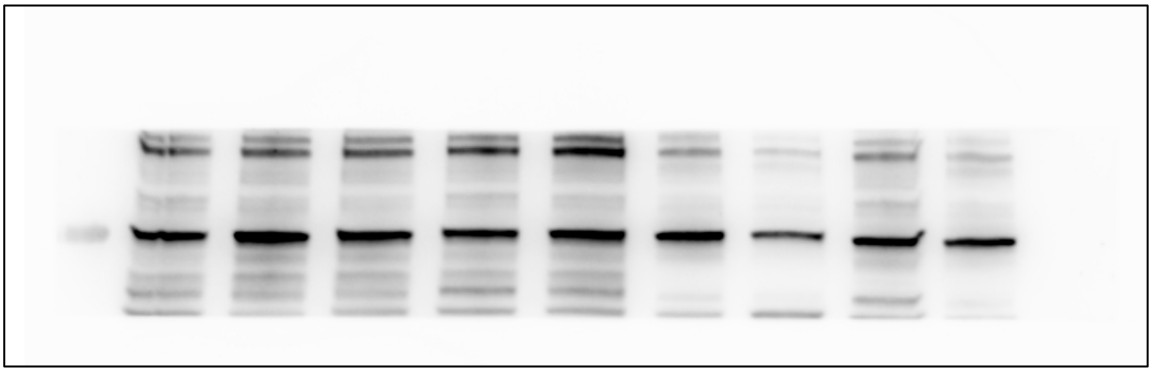

VIM

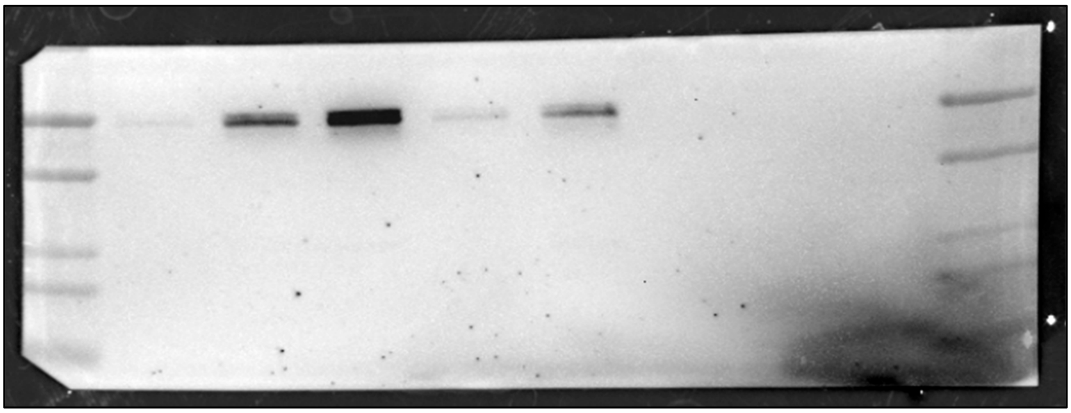

FLOT-1

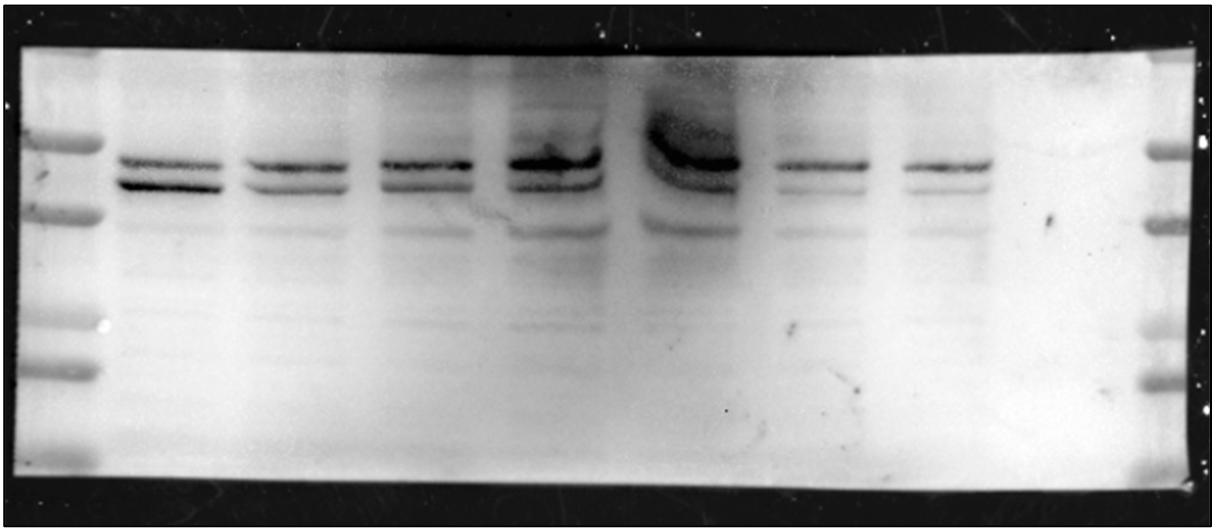

GAPDH

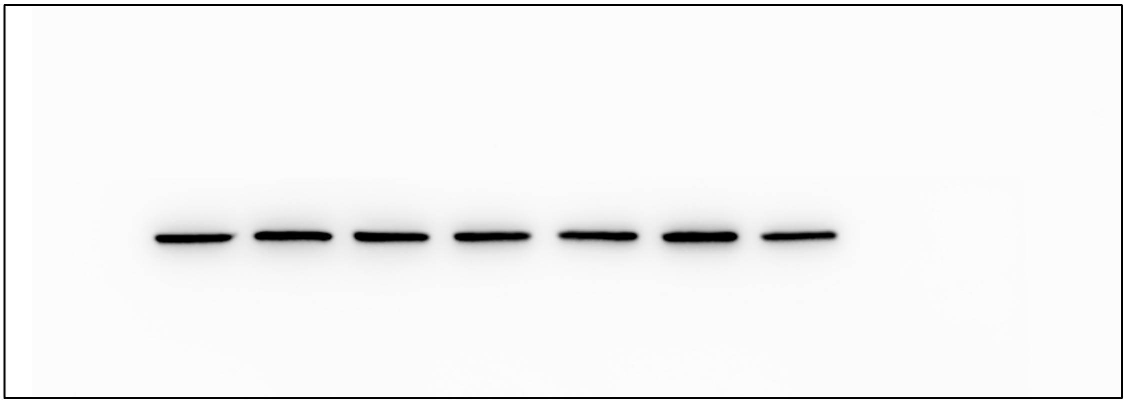

Figure 8-E

PDGFRβ

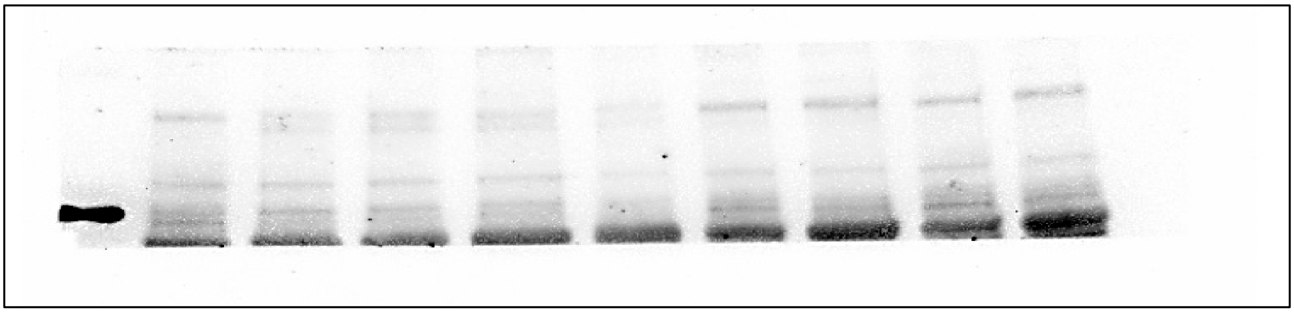

PPARγ

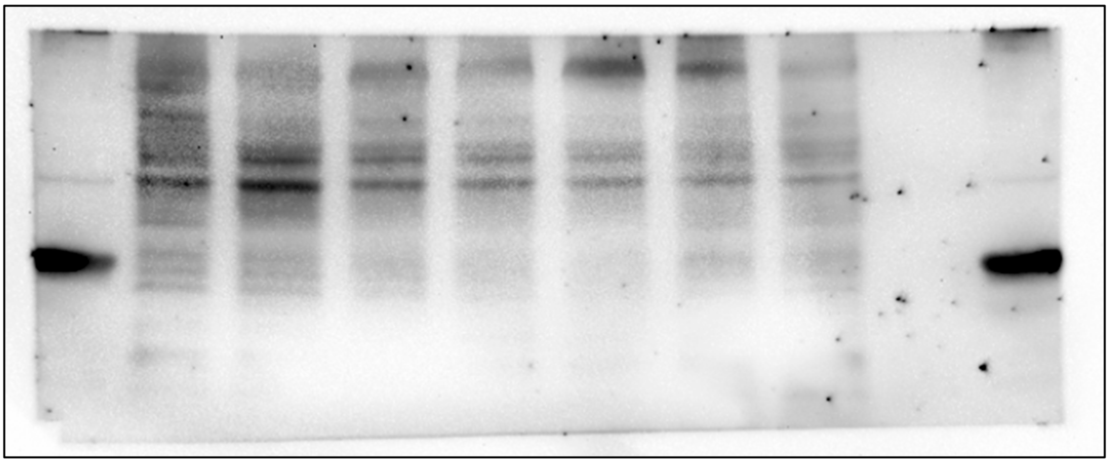

MDR1 (P-GP)

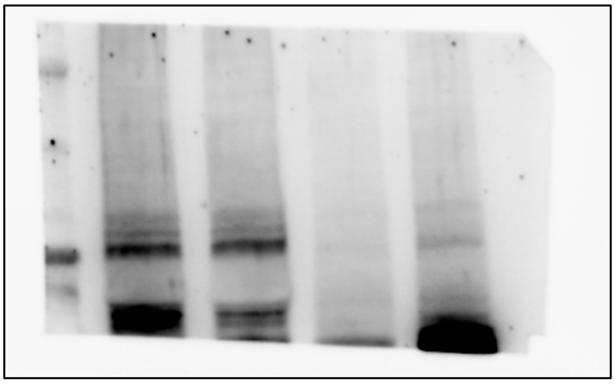

ANG-2

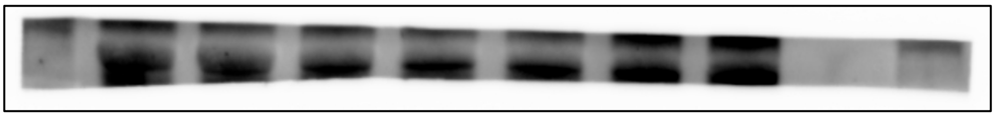

VEGFA

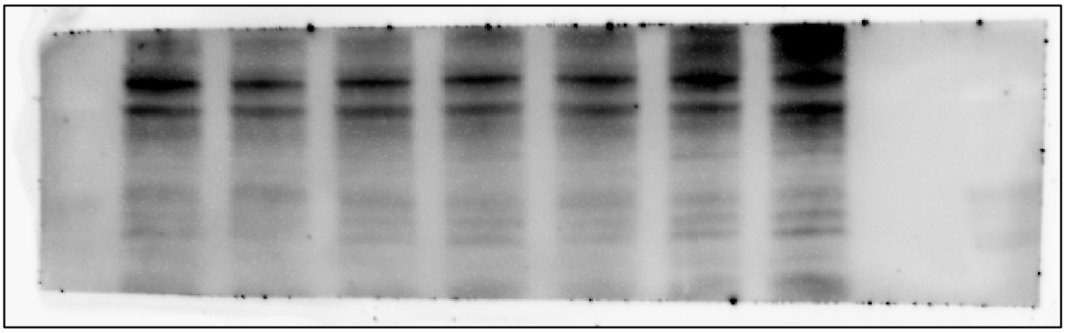

COL1A1

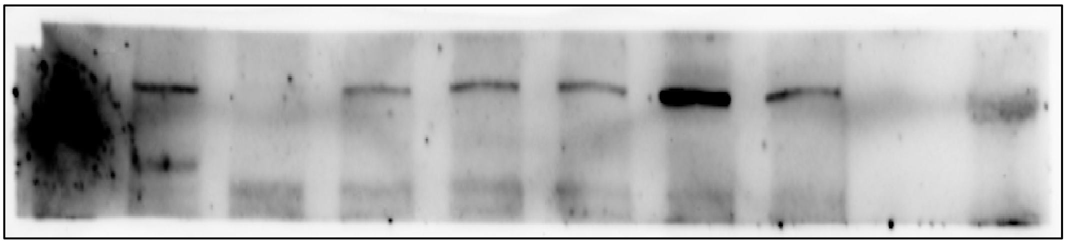

E-CAD

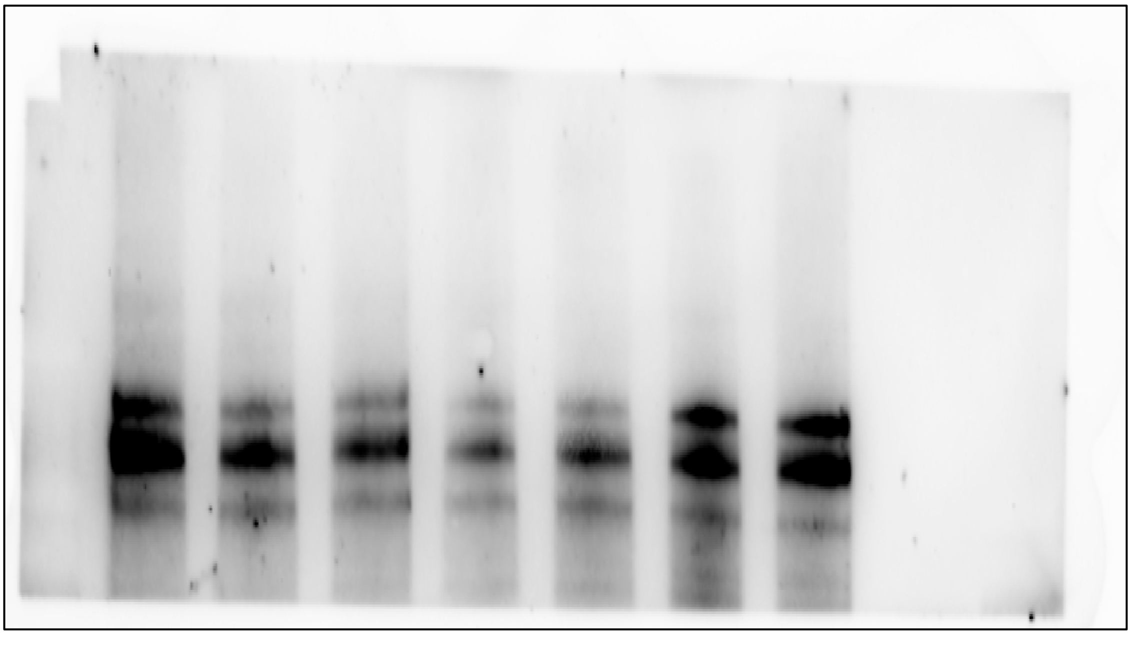

N-CAD

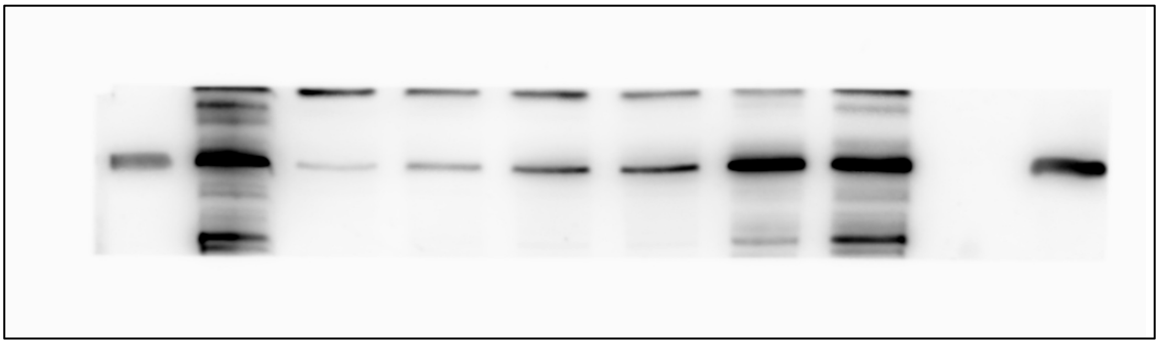

VIM

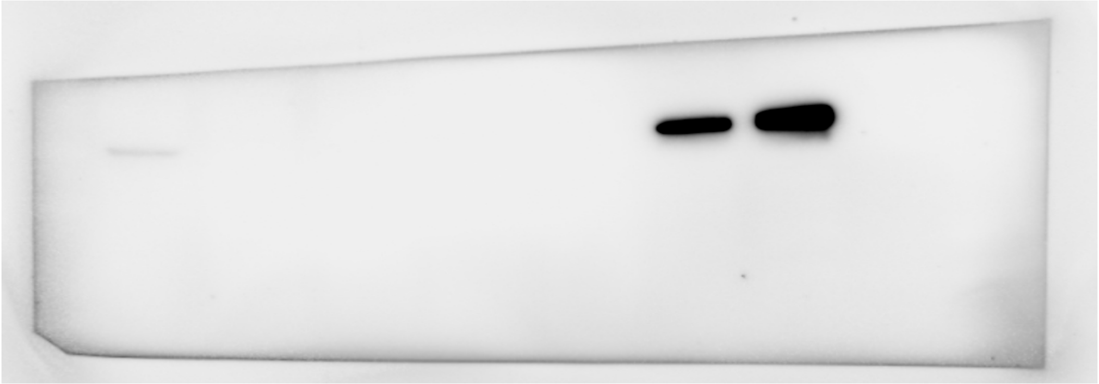

FLOT-1

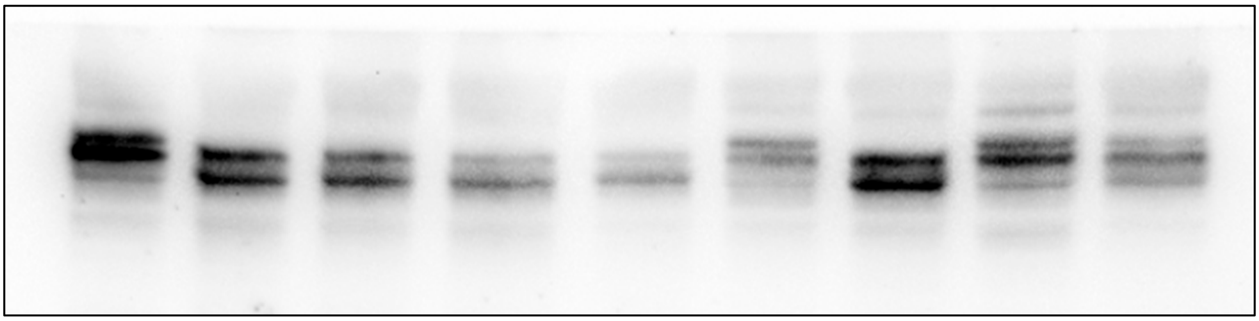

GAPDH

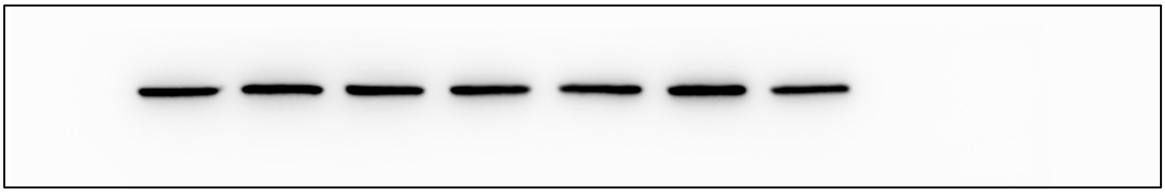

Supplement: Supplementary file 1 [file cancers-17-01360-s001.zip › cancers-3531505-supplementary.pdf]
